# Patency, Pregnancy, and Live Birth Outcomes of Microsurgical Vasovasostomy Compared with Vasoepididymostomy for Vasectomy Reversal: A Systematic Review and Meta-Analysis

**DOI:** 10.1590/S1677-5538.IBJU.2026.0353

**Published:** 2026-07-30

**Authors:** Azza Fithra Alhanifa, Putu Thio Mahapradana, Putu Krisna Agastya Artawan, Made Oka Negara, Gede Wirya Kusuma Duarsa

**Affiliations:** 1 Universitas Udayana Faculty of Medicine School of Medicine Denpasar Indonesia School of Medicine, Faculty of Medicine, Universitas Udayana, Denpasar, Indonesia; 2 Universitas Udayana Faculty of Medicine Department of Andrology and Sexology Denpasar Indonesia Department of Andrology and Sexology, Faculty of Medicine, Universitas Udayana, Denpasar, Indonesia; 3 Universitas Udayana Faculty of Medicine Department of Urology Denpasar Indonesia Department of Urology, Faculty of Medicine, Universitas Udayana, Denpasar, Indonesia

**Keywords:** Vasovasostomy, Azoospermia, Pregnancy Rate

## Abstract

**Purpose:**

To compare patency, pregnancy, and overall live-birth outcomes after microsurgical vasovasostomy (VV) versus vasoepididymostomy (VE), and to evaluate mixed bilateral procedures as a distinct analytic category.

**Materials and Methods:**

A systematic search of four databases was conducted. Eligible studies were observational cohorts reporting patency, pregnancy, or overall live-birth outcomes of men undergoing microsurgical VV, VE, or mixed bilateral reversal for post-vasectomy obstructive azoospermia. The quality of studies was assessed using the Newcastle-Ottawa Scale. Random-effects meta-analysis using restricted maximum likelihood was performed in R, reporting pooled odds ratios (OR) for dichotomous outcomes and mean differences (MD) for continuous outcomes, both with 95% confidence intervals (CI). Heterogeneity was quantified using I^2^. The protocol was registered in PROSPERO (CRD420261357366).

**Results:**

Thirteen studies encompassing 6,867 patients (4,053 VV; 1,430 VE; 1,384 mixed) were included. VV was associated with significantly higher patency than VE (OR 6.98, 95% CI 4.23 to 11.50; I^2^=52.6%), higher natural pregnancy odds (OR 2.01, 95% CI 1.38 to 2.91), higher overall live-birth odds (OR 2.79, 95% CI 1.67 to 4.67), and earlier sperm return (MD −1.95 months). Mixed procedures yielded intermediate patency. Findings were robust across all sensitivity analyses; small-study effects could not be fully excluded given the limited number of studies contributing to some outcomes.

**Conclusions:**

VV is associated with higher patency, pregnancy, and overall live-birth rates than VE, reflecting more favorable anatomical conditions. Mixed bilateral procedures yield intermediate outcomes from asymmetric anatomy. These findings support VV and VE as complementary techniques selected according to intraoperative vasal fluid findings.

## INTRODUCTION

Vasectomy is the most common sterilization procedure worldwide, and approximately 6% of patients later seek reversal because of remarriage, desire for more children, or post-vasectomy pain (1). Microsurgical vasovasostomy (VV) remains the leading technique for reversing vasectomy-induced obstruction, restoring fertility directly and costing less than sperm retrieval with intracytoplasmic sperm injection (ICSI) (2, 3).

The two main techniques are VV, which directly reconnects the severed vas deferens, and vasoepididymostomy (VE), which bypasses secondary epididymal obstruction by connecting the vas to an epididymal tubule (4). Vasal fluid quality guides intraoperative selection; abundant sperm-containing fluid favors VV, whereas thick, sperm-free fluid requires VE (5, 6). Frequently, asymmetrical intraoperative findings require a mixed bilateral reconstruction, defined as unilateral VE performed concurrently with contralateral VV.

Separate meta-analyses have reported patency rates for VV and VE (7, 8). Pregnancy outcomes vary with obstructive interval, female partner age, and unilateral reconstruction (6). Other studies have assessed sperm return and postoperative failure for each procedure separately (9). However, no previous meta-analysis has synthesized the comparative patency of VV and VE within the same cohorts, nor has any evaluated the effect of using both procedures bilaterally as a distinct analysis group.

This systematic review and meta-analysis compared microsurgical VV and VE in terms of five clinical parameters: overall patency rates, natural pregnancy rates, overall live-birth rates, time to patency, and late failure in patients who underwent vasectomy reversal (VR) for post-vasectomy obstructive azoospermia. We also evaluated mixed bilateral surgical technique as a distinct analysis group.

## MATERIALS AND METHODS

### Search Strategy

The present systematic review and meta-analysis were conducted according to the Preferred Reporting Items for Systematic Reviews and Meta-Analyses (PRISMA) 2020 checklist (10). Databases including PubMed, Scopus, Europe PMC, and Epistemonikos were accessed from their inception until 31 March 2026 without language limitations. Search strategy was performed based on the Population, Intervention, Comparison, Outcomes, and Study design (PICOS) principle, using controlled vocabulary terms and free text key words for VR, such as vasovasostomy, vasoepididymostomy, and related synonyms (e.g., "vas deferens reversal," "epididymovasostomy"). We applied Boolean operators (AND, OR) to link search terms systematically across all databases. All the exact Boolean search strings used are available in Supplementary Material (see APPENDIX 1). Unpublished or grey literature was sought through registries and conference proceedings. Additionally, studies were manually screened to identify additional eligible studies.

### Eligibility Criteria

Eligibility criteria followed the PICOS methodology. We included adult males undergoing microsurgical VR for obstructive azoospermia secondary to vasectomy. The comparison was microsurgical VV versus VE, whether unilateral, bilateral, or mixed combination technique. The primary outcome of interest was the overall patency rate. For the purposes of this review, anatomical patency was defined as the presence of any spermatozoa, whether motile or immotile, in the ejaculate following surgery. Functional patency was defined on the basis of motile spermatozoa or specified motile sperm concentrations, according to the thresholds reported or prespecified by the original study authors. Secondary outcomes included natural pregnancy rate, overall live birth rate, time to patency, and late failure after initial sperm return. Eligible designs were randomized controlled trials and cohorts.

We excluded studies of non-vasectomy obstruction without stratified data and non-comparative reports, including case reports and case series. We also excluded reviews, editorials, abstracts, animal studies, and reports without accessible full text. Duplicate records were removed before screening. Two reviewers (IPTM and IPKAA) conducted the screening independently.

### Data Extraction

Two reviewers (IPTM and IPKAA) independently extracted data using a structured form; disagreements were resolved by consensus with AFA, IMON, and/or GWKD. Extracted variables included publication year, country, study design, sample size, procedure types (VV/VE), surgical techniques, vasal fluid quality, female partner age, follow-up duration, previous reversal failures, patency definitions, selection criteria, pregnancy type (ART/natural), patent-only denominators, and clinical outcomes.

### Quality Assessment

Methodological quality of included non-randomized studies was evaluated using the Newcastle-Ottawa Scale (NOS). The studies were later classified into good, fair, or poor quality based on the criteria established by the Agency for Healthcare Research and Quality (AHRQ). Two reviewers (IMON and GWKD) independently rated each study; disagreements were resolved by a third reviewer (AFA).

## Statistical Analyses

All analyses were performed in R, with statistical significance set at p < 0.05. Pooled estimates used random-effects models with restricted maximum likelihood. Categorical data were presented as pooled unadjusted odds ratios (OR), whereas continuous data were presented as pooled mean differences (MD), both with 95% confidence intervals (CI). Although several primary studies reported multivariable analyses using heterogeneous covariate sets, adjusted estimates were not consistently available for the VV-versus-VE comparison and could not be harmonized; therefore, an adjusted-estimate-only sensitivity analysis was not feasible. Aggregated crude event proportions across contributing studies were also calculated by summing events and denominators per group. Heterogeneity was assessed with I^2^ and classified as low (0-25%), moderate (26-50%), substantial (51-75%), or considerable (>75%). Pregnancy and overall live birth outcomes were analyzed across patients with available reproductive follow-up and, as a post hoc exploratory analysis, restricted to patent cases; natural pregnancy rates were analyzed separately from ART-inclusive rates, with the ART-inclusive analysis also considered post hoc, as it was not prespecified in the study protocol. Subgroup analyses examined differences by surgery type, patency definition, VE technique, and intraoperative selection criteria. Exploratory post-hoc meta-regression assessed the influence of female partner age, follow-up duration, and prior failed reversal proportion. Sensitivity analyses excluded high-risk-of-bias studies, applied leave-one-out analysis, and removed the largest cohorts. Publication bias was assessed using Egger's test. Certainty of evidence for each outcome was assessed using the Grading of Recommendations Assessment, Development, and Evaluation (GRADE) framework.

### Ethical Approval and Registration

In line with The Council for International Organizations of Medical Sciences (CIOMS) Guideline 23, studies using publicly available data are exempt from ethics review (11). The protocol was prospectively registered in PROSPERO (CRD420261357366).

## RESULTS

### Study Selection and Characteristics

A systematic search of four databases identified 4,115 records, of which 2,142 unique records underwent title and abstract screening after duplicate removal (Figure-1). Of 2,142 screened records, 2,122 were excluded. Twenty full texts were sought; two were unavailable and five were excluded for non-vasectomy obstruction, leaving 13 studies (6, 12–23). All were observational (twelve retrospective cohorts, one multicenter prospective cohort), encompassing 6,867 patients: 4,053 VV, 1,430 VE, and 1,384 mixed bilateral procedures. Mean male age ranged from 37 to 47 years, female partner age from 29 to 34 years, and obstructive interval from 7.0 to 18.0 years. All procedures used microsurgery. VV techniques included two-layer, modified one-layer, multilayer, and microdot approaches; VE techniques included end-toside, end-to-end, intussusception, and combined approaches. One study exclusively enrolled repeat-reversal patients (Table-1).

**Figure 1:**
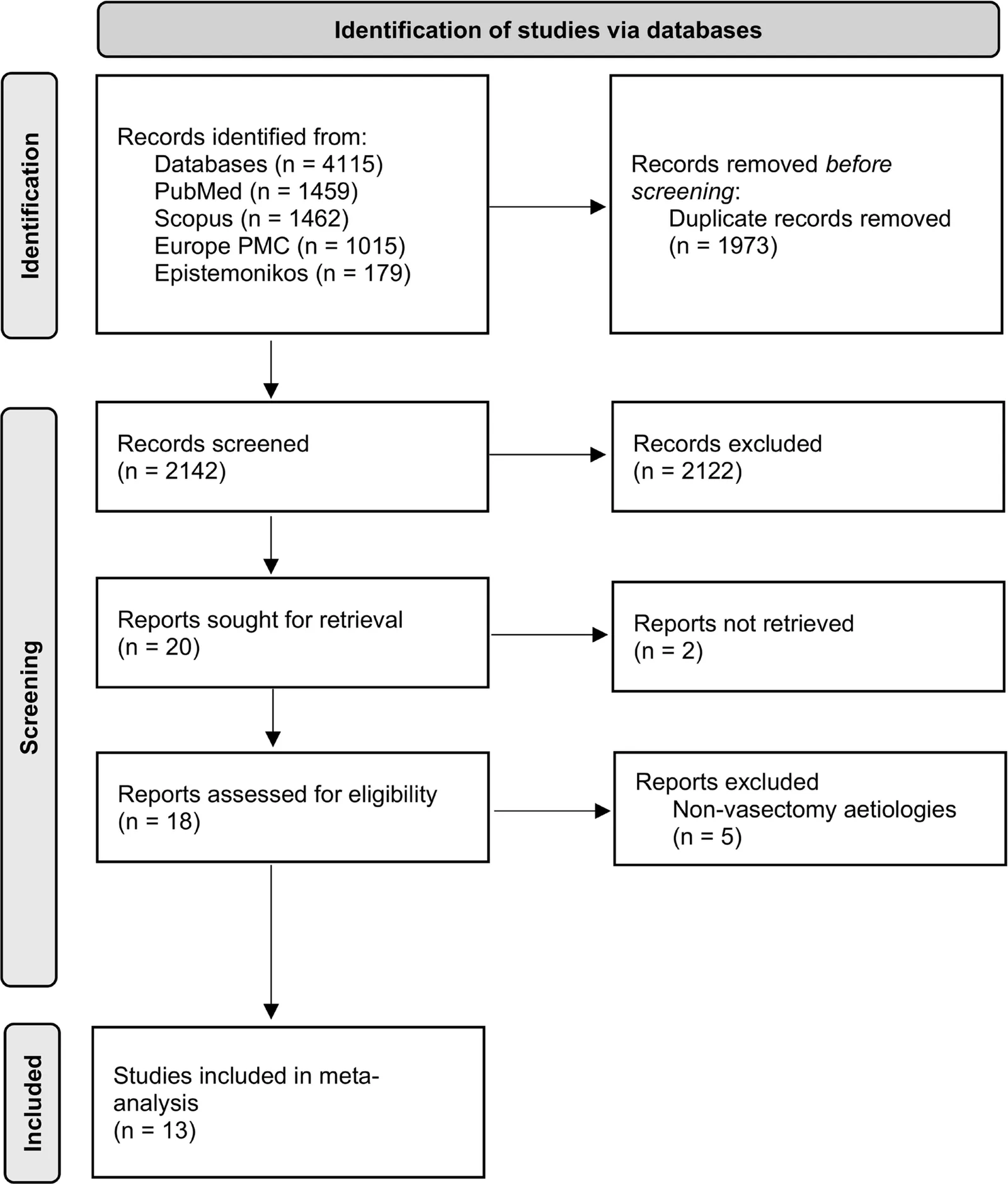
PRISMA Flow Diagram.

Overview of the literature search, study screening, and selection process for the systematic review and meta-analysis.

**Table 1:** Baseline characteristics of included studies.

Study	Country	Study Design	Total N	VV, n	VE, n	Mixed, n	Mean Male Age, y	Mean Female Partner Age, y	Mean OI, y	VV Anastomosis Technique	VE Anastomosis Technique	Previous Failed Reversal %
**Alhamam et al., 2024** (19)	Canada	Retrospective cohort	136	101	12	23	41.0 ± 6.2	34.0 ± 4.6	8.0 ± 5.5	Microdot 2-layer	Longitudinal intussusception (LIVE)	NR
**Belker et al., 1991** (6)	USA	Multicenter prospective cohort	1,158	1,061	38	59	36.9 (range 20.3–67.4)	29.6 (range 17.0– 46.0)	7.0 (range <1.0– 33.0)	2-layer or modified 1-layer	End-to-end or end-to-side	15.1%
**Boorjian et al., 2004** (21)	USA	Retrospective cohort	213	159	36	18	NR	NR	NR	Multilayer	Multilayer	0%
**Fuchs and Burt, 2002** (15)	USA	Retrospective cohort	116	44	42	30	46.6†	34.3	18.0	2-layer	End-to-side	NR
**Hertz et al., 2021** (18)	USA	Retrospective cohort	423	385	16	22	36.6 (range 23–76)	31.0 (range 19–46)	7.9 (range 0–34)	Modified 2-layer	End-to-side intussusception	6.5%
**Magheli et al., 2010** (22)	Germany	Retrospective cohort	312	242	24	46	41.1 ± 6.7	31.6 ± 5.1	NR	Multilayer	NR	NR
**Maloney et al., 2026** (16)	USA	Retrospective cohort	326	248	33	45	38.4 ± 5.8 (VV); 40.0 ± 6.2 (Mixed); 40.4 ± 7.4 (VE)	NR	7.8 ± 4.5 (VV); 10.9 ± 4.5 (Mixed); 12.4 ± 5.6 (VE)	2-layer	Intussusception	9.0%
**Matthews et al., 1995** (12)	USA	Retrospective cohort	200	100	100	0	NR	NR	NR	Multilayer	End-to-end (n=85); end-to-side (n=15)	NR
**Matthews et al., 1997**‡ (13)	USA	Retrospective cohort	64	28	27	9	NR	NR	NR§	Multilayer	End-to-side or end-to-end	100%
**Schrepferman et al., 2001** (23)	USA	Retrospective cohort	84	46	18	20	NR	NR	NR	2-layer	End-to-side	NR
**Schwarzer et al., 2004** (14)	Germany	Retrospective cohort	307	192	48	67	42.0 (range 24–67)	NR	8.0 (range 0–27)	3-layer, end-to-end	End-to-side, 2–3-layer	12.0%
**Silber & Grotjan, 2004** (20)	USA	Retrospective cohort	3,378	1,357	1,013	1,008	40.0 ± 7.1	31.0 ± 5.0	10.0 ± 5.4	2-layer	End-to-end or end-to-side	22.3%
**Yang et al., 2007** (17)	USA	Retrospective cohort	150	90	23	37	42.9 (range 27–61)	NR	11.1 (range 1–29)	2-layer	4 or 6-point invagination	NR

VV = vasovasostomy; VE = vasoepididymostomy; Mixed = bilateral procedure with VV on one side and VE on the other; OI = obstructive interval (time since vasectomy); NR = not reported; y = years; n = number.

† Median value reported.

‡ Repeat reversal cohort (all patients had prior failed vasectomy reversal).

§ Interval from vasectomy to initial reversal: 10.7 ± 5.2 years (complete failures), 8.7 ± 5.5 years (late failures).

All procedures were performed using microsurgical techniques. Data are presented as mean ± SD, mean (range), or n/N (%) where available

### Quality Assessment

Nine studies were rated good quality and four poor quality (Table-2). Poor ratings reflected unadjusted baseline differences in comparability domains. Good quality studies included five that utilized multivariable regressions and four using stratified bivariate analyses based on obstruction periods. Regardless of quality, the major limitation in all studies was insufficient patient follow-up in pregnancy outcomes, even among the two largest samples, which lost more than 20% of patients for fertility analysis.

**Table 2:** Newcastle-Ottawa scale quality assessment of studies.

Study	Selection	Comparability	Outcome	Quality	AHRQ classification
**Alhamam et al., 2024** (19)	2	0	3	5	Poor quality
**Belker et al., 1991** (6)	3	1	2	6	Good quality
**Boorjian et al., 2004** (21)	4	2	2	8	Good quality
**Hertz et al., 2021** (18)	4	2	3	9	Good quality
**Magheli et al., 2010** (22)	3	2	2	7	Good quality
**Fuchs & Burt, 2002** (15)	2	0	3	5	Poor quality
**Maloney et al., 2026** (16)	3	2	2	7	Good quality
**Matthews et al., 1995** (12)	4	1	2	7	Good quality
**Matthews et al., 1997** (13)	4	1	3	8	Good quality
**Schrepferman et al., 2001** (23)	2	0	3	5	Poor quality
**Schwarzer et al., 2004** (14)	3	1	1	5	Poor quality
**Silber & Grotjan, 2004** (20)	4	1	2	7	Good quality
**Yang et al., 2007** (17)	4	1	2	7	Good quality

Agency for Healthcare Research and Quality.

### Overall Patency Rate

Aggregated event proportions for clinical outcomes are summarized in Table-3. The overall patency rate was significantly higher following VV (91.6%, 3714/4053) compared to VE (74.7%, 1069/1430) (OR 6.98, 95% CI 4.23 to 11.50; P < 0.0001; I^2^ = 52.6%) (Figure-2A). The advantage of VV over mixed procedures was smaller, yet it remained significant (OR 2.02, 95% CI 1.29 to 3.17; P = 0.0022; I^2^ = 49.4%) (Figure-2B). At the same time, mixed procedures (91.1%, 1261/1384) resulted in significantly higher patency than VE alone (75.5%, 1004/1330) (OR 3.52, 95% CI 2.78 to 4.45; P < 0.0001; I^2^ = 0.0%) (Figure-2C). In two studies, sperm returned earlier after VV (MD −1.95 months; 95% CI −2.59 to −1.31; P < 0.0001; I2 = 0.0%).

**Table 3:** Summary of aggregated event proportions by surgical technique.

Clinical Outcome	Vasovasostomy (VV)	Vasoepididymostomy (VE)	Mixed Bilateral (VV/VE)
**Overall Patency Rate**	91.6% (3714/4053)	74.7% (1069/1430)	91.1% (1261/1384)
**Natural Pregnancy Rate**†	69.0% (1636/2372)	65.5% (461/704)	—
**Natural Pregnancy Rate (Patent-Only)**	64.8% (476/734)	54.1% (98/181)	—
**ART-Inclusive Pregnancy Rate**† *	74.9% (1225/1635)	67.2% (420/625)	—
ART-Inclusive Pregnancy Rate (Patent-Only)*	86.9% (835/960)	81.0% (350/432)	—
**Overall Live Birth Rate**†	78.4% (551/703)	58.2% (258/443)	—
**Overall Live Birth Rate (Patent-Only)**	78.7% (483/614)	69.4% (218/314)	—

VV = vasovasostomy; VE = vasoepididymostomy; Mixed = bilateral procedure with VV on one side and VE on the other.

* Overall pregnancy includes pregnancies achieved via natural conception and Assisted Reproductive Technology (ART).

† All patients with available reproductive follow-up.

**Figure 2A:**
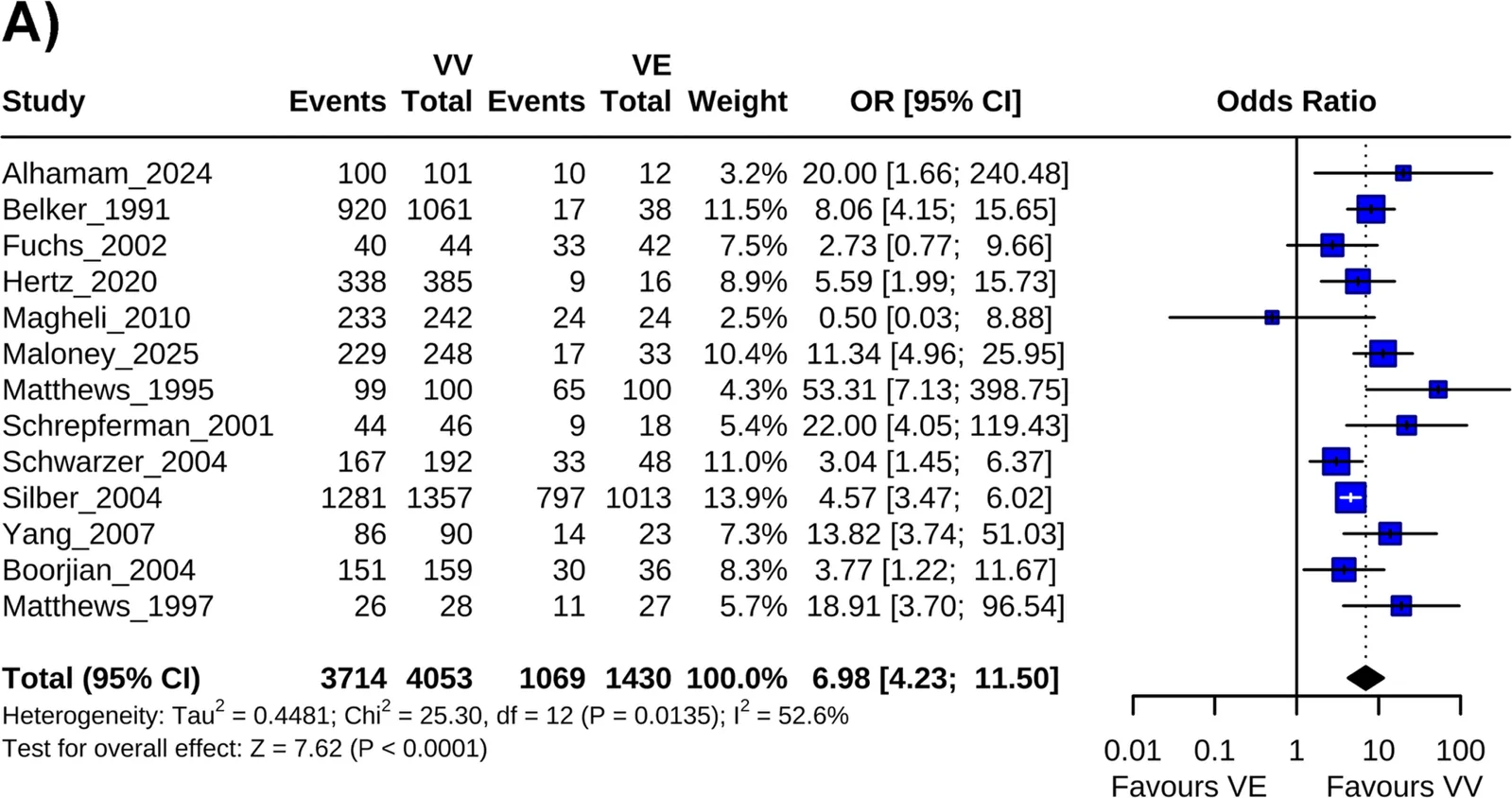
Forest plots of overall patency rates.

**Figure 2B:**
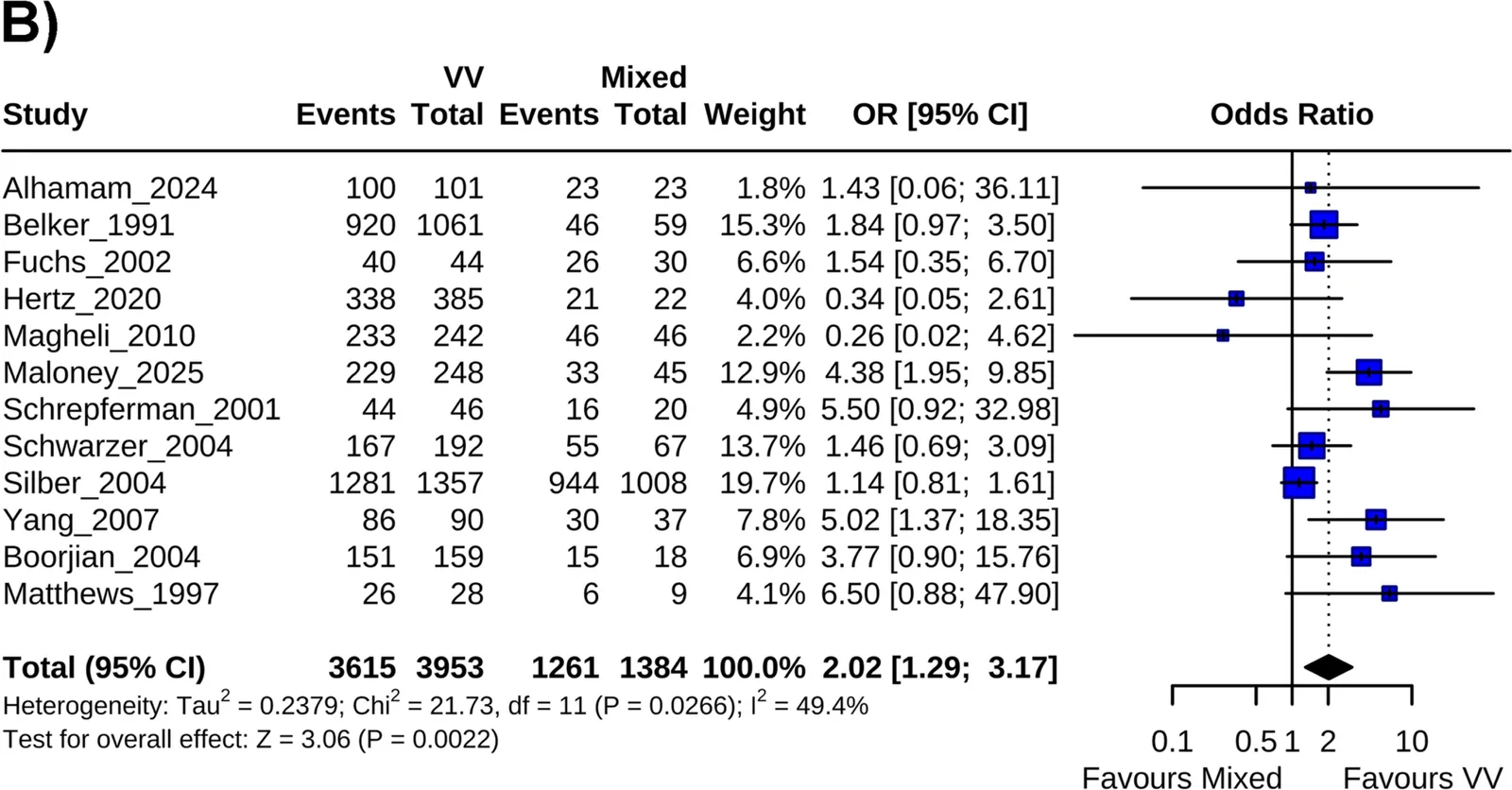
Forest plots of overall patency rates.

**Figure 2C:**
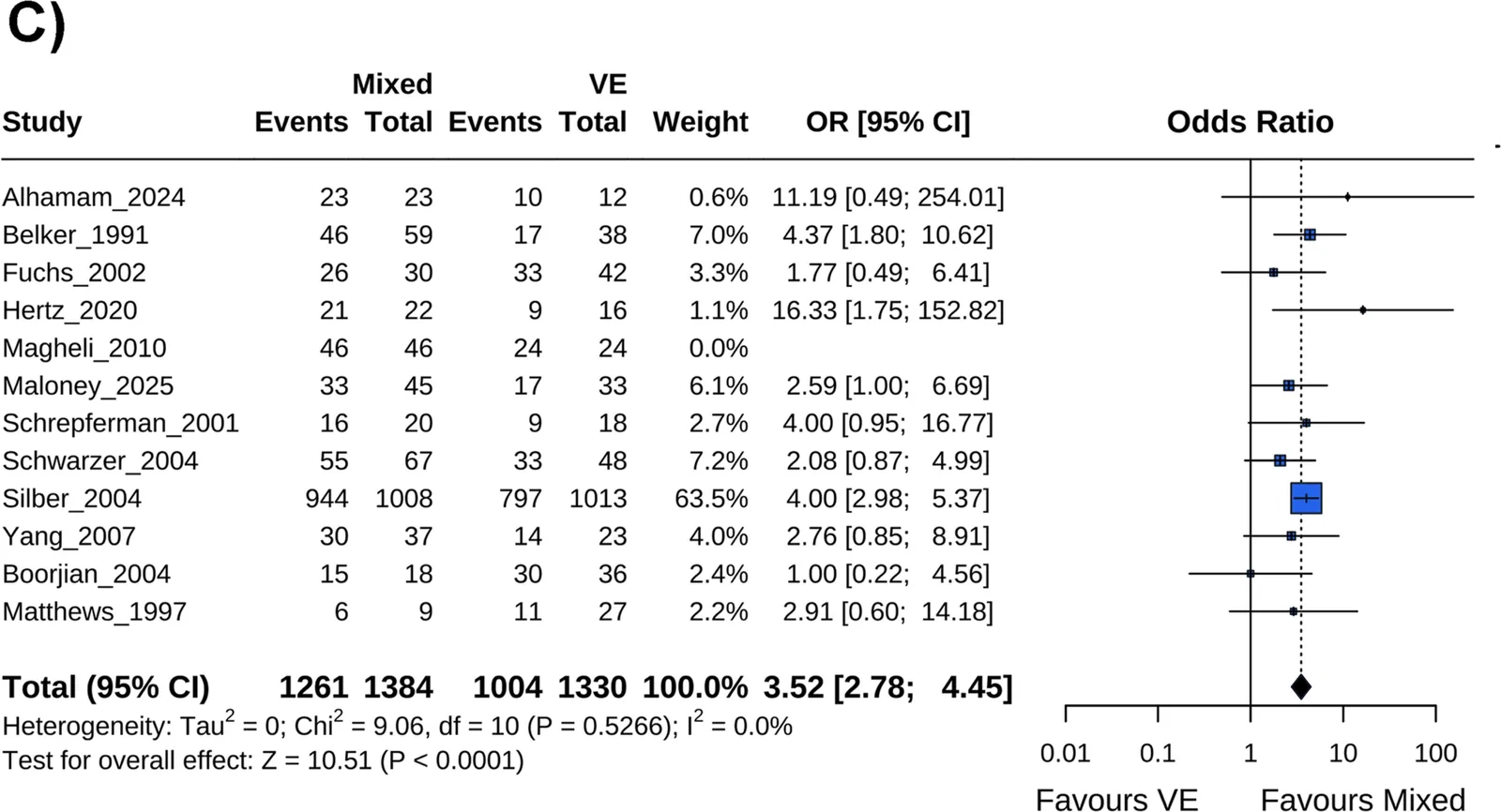
Forest plots of overall patency rates.

Comparison of postoperative patency outcomes across surgical techniques. A) Microsurgical vasovasostomy (VV) versus vasoepididymostomy (VE). B) Isolated VV versus mixed bilateral procedures. C) Isolated mixed bilateral procedures versus VE.

### Pregnancy Rate and Overall Live Birth

Substantial loss to follow-up was noted for reproductive outcomes. Natural pregnancy occurred in 69.0% (1636/2372) of VV cases and 65.5% (461/704) of VE cases among patients with available reproductive follow-up. A detailed breakdown of patient attrition from initial surgery to final reproductive follow-up is provided in Supplementary Table-1, 2, and 3 (see APPENDIX 2). The pooled odds of natural pregnancy were higher after VV compared to VE (OR 2.01, 95% CI 1.38 to 2.91; P = 0.0002; I^2^ = 38.7%) (Figure-3A). Including pregnancies achieved through assisted reproductive technology (ART) similarly showed higher pooled odds in favor of VV (74.9%, 1225/1635) compared to VE (67.2%, 420/625) (OR 2.92, 95% CI 1.77 to 4.82; P < 0.0001; I^2^ = 51.6%). Overall live-birth data (including both natural and ART-assisted conceptions) were available from only three studies. Each of these studies defined this outcome simply as a pregnancy leading to a live birth or the delivery of a baby, confirming that comparable clinical endpoints were pooled. Results favored VV (78.4%, 551/703) over VE (58.2%, 258/443) (OR 2.79, 95% CI 1.67 to 4.67; P < 0.0001; I^2^ = 59.0%) (Figure-3B). Pooling late failure data (15.1%, 19/126 for VV vs. 16.5%, 13/79 for VE) resulted in a non-significant estimate with massive heterogeneity (OR 1.30, 95% CI 0.17 to 9.84; P = 0.80; I^2^ = 78.3%).

**Figure 3:**
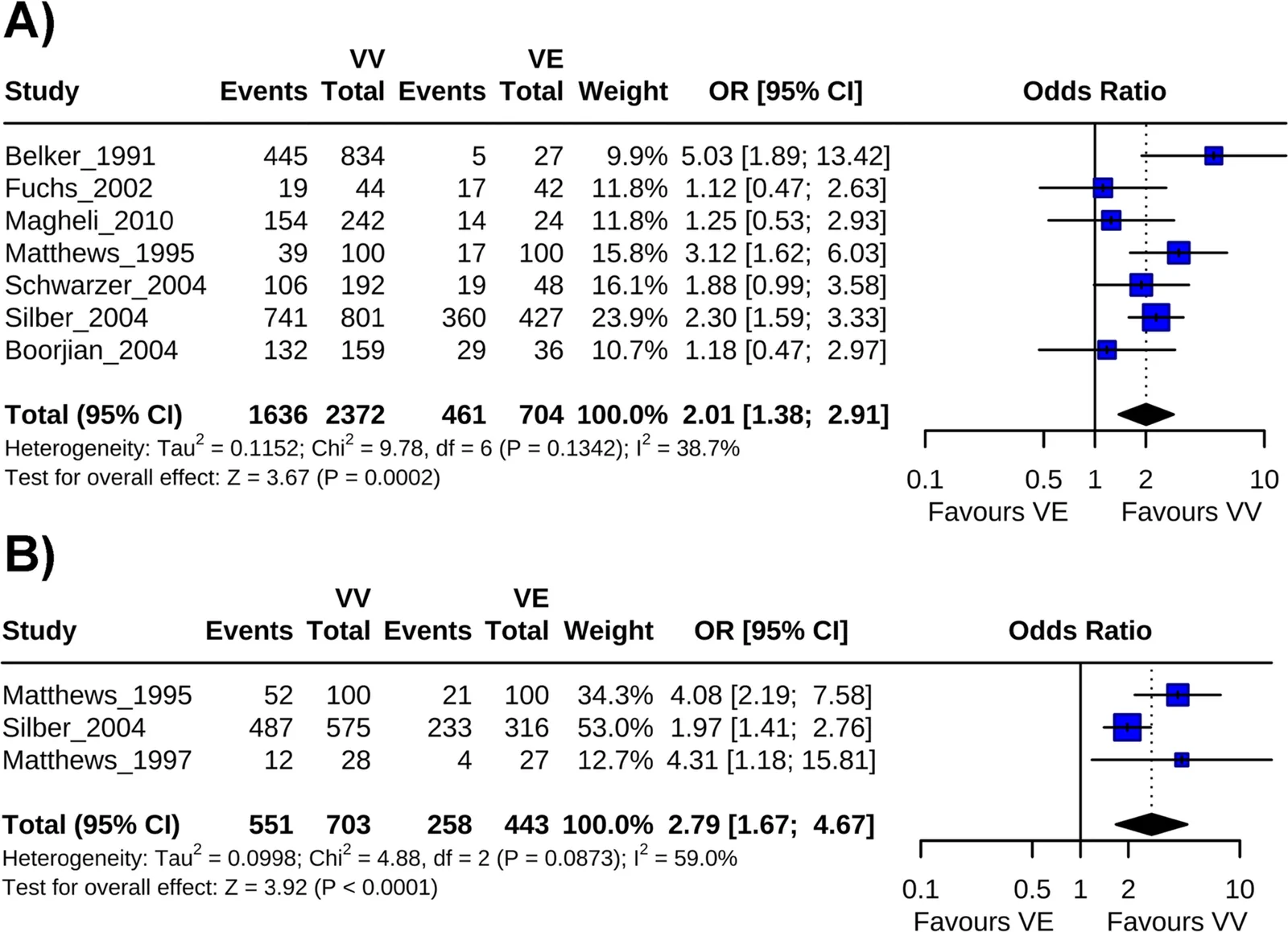
Forest plots of reproductive outcomes.

Comparison of reproductive success between patients undergoing microsurgical vasovasostomy (VV) versus vasoepididymostomy (VE). A) Natural pregnancy rates. B) Overall live birth rates (natural and ART-assisted conceptions combined).

In a post hoc analysis restricted to patent-only patients, the differences in reproductive outcomes between VV and VE were attenuated. After excluding non-patent patients, natural pregnancy (64.8%, 476/734 for VV vs. 54.1%, 98/181 for VE; OR 1.24, 95% CI 0.76 to 2.03; I^2^ = 15.5%) and ART-inclusive pregnancy (86.9%, 835/960 for VV vs. 81.0%, 350/432 for VE; OR 1.52, 95% CI 0.80 to 2.89; P = 0.201; I^2^ = 26.4%) were not significantly different between VV and VE groups. Thus, the reproductive difference associated with VV appears largely explained by greater patency; among men who achieve patency, couple-level factors may predominate. Overall live birth, however, remained higher after VV among patent patients (78.7%, 483/614 vs. 69.4%, 218/314; OR 1.73, 95% CI 1.25 to 2.41; P = 0.001; I^2^ = 0.0%).

### Subgroup, Meta-Regression, and Sensitivity Analyses

Stratification by VE technique showed that both intussusception (91.4%, 753/824 for VV vs. 59.5%, 50/84 for VE; OR 9.82, 95% CI 5.59 to 17.26; I^2^ = 0%) and traditional approaches (91.3%, 2684/2941 for VV vs. 75.6%, 986/1304 for VE; OR 5.92, 95% CI 3.04 to 11.54; I^2^ = 54.1%) significantly favored VV (Figure-4A). There was no between-subgroup difference (P = 0.26). Heterogeneity was absent in the intussusception subgroup and substantial among traditional technique studies. Subgroup analyses based on intraoperative criteria further demonstrated that VV consistently achieved higher patency compared with VE across varying sperm quality and fluid conditions (Figure-4B). In cases where intact sperm were present and selection was fluid-dependent, VV showed a pooled OR of 7.55 (95% CI 4.13 to 13.78; I^2^ = 60.1%) with a patency rate of 94.4% (1696/1796) compared to 77.5% (838/1081) for VE. Liberal VV selection and strict azoospermia subgroups also demonstrated higher patency for VV (86.7%, 920/1061 and 93.7%, 760/811, respectively) over VE (44.7%, 17/38 and 69.5%, 205/295, respectively), with ORs of 8.06 (95% CI 4.15 to 15.65; I^2^ not applicable for a single study) and 6.36 (95% CI 2.24 to 18.08; I^2^ = 62.8%), respectively. Stratification by patency definition showed no significant subgroup difference between anatomical (92.2%, 3013/3268 for VV vs. 76.4%, 960/1256 for VE; OR 7.27; I^2^ = 59.9%) and functional patency (90.1%, 534/593 for VV vs. 60.3%, 76/126 for VE; OR 8.56; I^2^ = 37.1%; P = 0.77) (Figure-4C).

**Figure 4:**
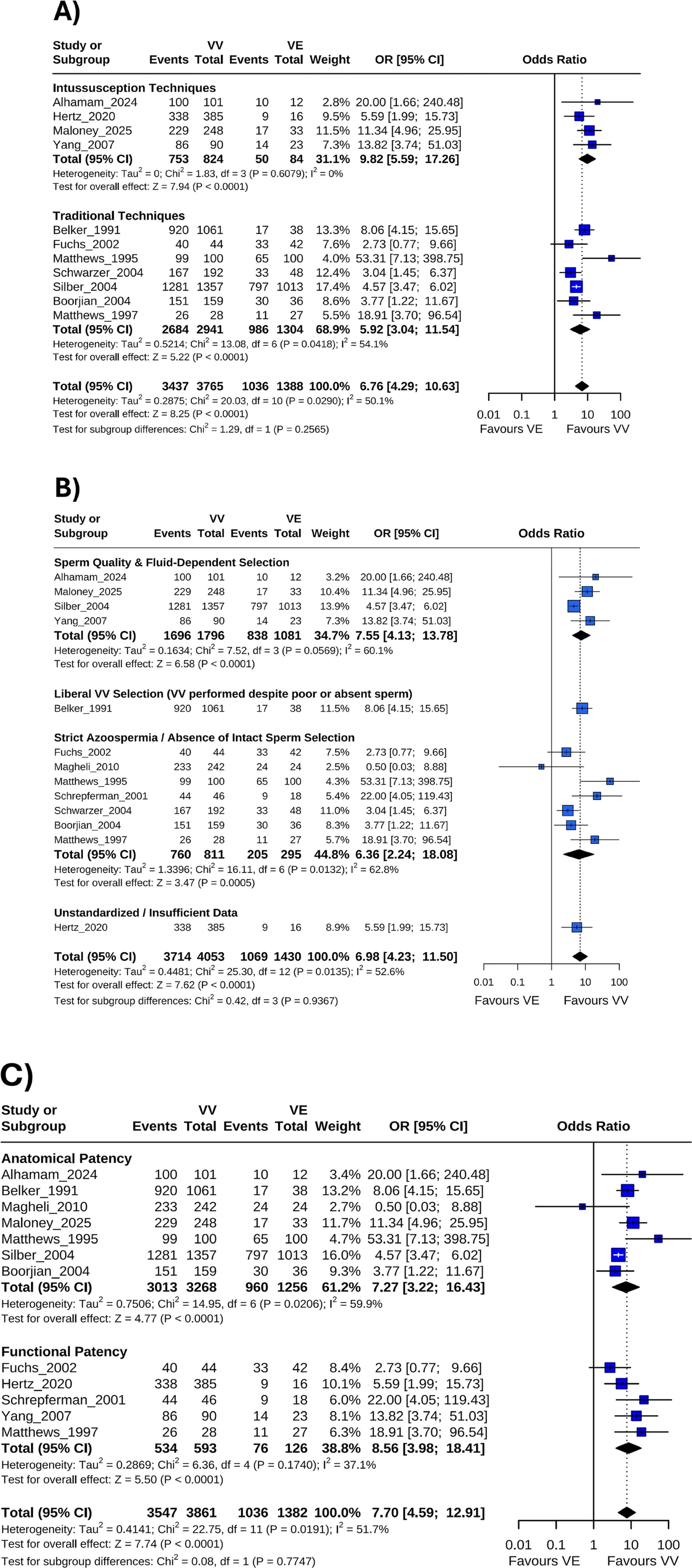
Forest plots of subgroup analyses for overall patency.

A) VE surgical technique (intussusception versus traditional approaches); B) intraoperative selection criteria (sperm quality and fluid-dependent selection, liberal VV selection, strict azoospermia/absence of intact sperm, and unstandardized data); and C) definition of patency (anatomical versus functional

Exploratory post-hoc meta-regression suggested that longer follow-up duration was associated with attenuation of the advantage of VV over VE for natural pregnancy (coefficient = −0.085, P = 0.014), supporting the view that pregnancy outcomes are time dependent. In contrast, follow-up duration was not a significant moderator for overall live birth or patency outcomes. Mean female partner age correlated negatively with natural pregnancy (coefficient = −0.283, P = 0.026). The proportion of prior failed reversals did not moderate patency or pregnancy outcomes in any comparison. Despite a clinically plausible positive coefficient, moderate-to-high residual heterogeneity suggests that vasal fluid quality, technique, and surgeon expertise may be more influential.

Leave-one-out analysis for the primary comparison of VV versus VE overall patency yielded OR estimates ranging from 6.25 to 7.70 (all P < 0.0001). Excluding the two largest cohorts (OR 7.52, 95% CI 3.97 to 14.23; I^2^ = 53.6%) or restricting the analysis to higher-quality studies (OR 7.65, 95% CI 4.24 to 13.79; I^2^ = 55.6%) did not change the direction or magnitude of the effect favoring VV over VE for patency outcomes.

### Publication Bias

Egger's test did not show funnel plot asymmetry for the main pairwise patency comparisons; VV vs VE (P = 0.15) supported by all 13 studies, and VV vs mixed (P = 0.26) and VE vs mixed (P = 0.46), each supported by 12 studies. However, formal assessment of small-study effects was underpowered for the reproductive outcomes, because fewer than ten studies contributed to natural pregnancy (7 studies) and overall live birth (3 studies); therefore, publication bias could not be reliably excluded for these outcomes.

### Evidence Certainty

Certainty of evidence was very low for all five outcomes (overall patency, natural pregnancy, overall live birth, time to patency, and late failure), representing the observational, non-randomized design of all included studies; substantial confounding by indication inherent to intraoperative technique selection; and, for the reproductive outcomes, considerable missing outcome data. The Summary of Findings table (Table-4) presents relative and absolute effects with certainty ratings and supporting footnotes for each outcome.

**Table 4:** Summary of findings for microsurgical vasovasostomy versus vasoepididymostomy in adult men with post-vasectomy obstructive azoospermia: Key outcomes, absolute and relative effects per 1000 patients, and GRADE certainty ratings.

Outcome	Absolute difference with VV per 1000 patients (95% CI)	VE per 1000	Relative effect (95% CI)	Participants (studies)	Certainty (GRADE)	Comments
**Overall patency**	207 more (179 to 224 more)	747	OR 6.98 (4.23 to 11.50)	5483 (13 observational)	VERY LOW ⊕◯◯◯	Serious risk of bias; confounding by indication*
**Natural pregnancy**	137 more (69 to 192 more)	655	OR 2.01 (1.38 to 2.91)	3076 (7 observational)	VERY LOW ⊕◯◯◯	Very serious risk of bias and missing data†
**Overall live birth**	213 more (117 to 285 more)	582	OR 2.79 (1.67 to 4.67)	1146 (3 observational)	VERY LOW ⊕◯◯◯	Very serious risk of bias, inconsistency, and indirectness‡
**Time to patency**	1.95 months earlier (−2.59 to −1.31 months)	-	MD −1.95 (−2.59 to −1.31)	313 (2 observational)	VERY LOW ⊕◯◯◯	Serious risk of bias and imprecision§
**Late failure**	39 more (132 fewer to 495 more)	165	OR 1.30 (0.17 to 9.84)	205 (2 observational)	VERY LOW ⊕◯◯◯	Very serious inconsistency and imprecision¶

* Certainty started at LOW (observational studies) and was downgraded for risk of bias (crude/unadjusted estimates; confounding by indication inherent to intraoperative VV/VE selection). Large effect upgrade not applied because confounding by indication plausibly explains the magnitude observed, yielding a VERY LOW certainty rating.

† Certainty started at LOW (observational studies) and was downgraded to two levels for risk of bias (crude estimates, confounding by indication, and ~60% missing outcome data) and one level for dissemination bias. No factors met the criteria for upgrading, yielding a VERY LOW certainty rating.

‡ Certainty started at LOW (observational studies) and was downgraded for risk of bias (~73% missing outcome data), inconsistency (I²=59%, discordant point estimates), indirectness (non-uniform live-birth definitions), imprecision, and dissemination bias. No factors met the criteria for upgrading, yielding a VERY LOW certainty rating.

§ Certainty started at LOW (observational studies) and was downgraded due to serious risk of bias, serious imprecision (only 2 studies, N=313), and dissemination bias. No factors met the criteria for upgrading, yielding a VERY LOW certainty rating.

Certainty started at LOW (observational studies) and was downgraded due to serious risk of bias, very serious inconsistency (opposite-direction point estimates), very serious imprecision (CI spans large effects in both directions), and dissemination bias. No factors met the criteria for upgrading, yielding a VERY LOW certainty rating.

CI = Confidence interval; OR = Odds ratio; MD = Mean difference; VV = Vasovasostomy; VE = Vasoepididymostomy; GRADE = Grading of Recommendation Assessment, Development, and Evaluation.

## DISCUSSION

This current meta-analysis of 13 observational studies and 6,867 patients showed higher overall patency rates for VV compared with VE for VR (OR 6.98; 95% CI 4.23 to 11.50). VV patency in our analysis (91.6%) was comparable to pooled estimates of 87.5% for microsurgical VV and 94.4% for robot-assisted VV reported by Seth et al. (24), as well as 89.4% for microsurgical VV reported by Herrel et al. (25). Biologically, these higher patency rates reflect the anatomical reliability of suturing the thicker vas deferens wall, compared to the fragile VE anastomosis on a delicate epididymal duct (26). At the same time, 74.7% (1069/1430) patency of VE is close to the range of 64 to 70% obtained in intussusceptionrelated systematic reviews (7, 8). Consistent leave-one-out, large-cohort-exclusion, and quality-restricted analyses, together with no detected funnel-plot asymmetry, support the direction of effect, although residual confounding and heterogeneity remain.

Crucially, the observed differences in outcomes between VV and VE must be interpreted through the lens of confounding by indication and obstruction severity. VV and VE are not simply competing options for a uniform clinical scenario; rather, they are complementary techniques applied to different anatomical and biological conditions. VV is preferentially performed in more favorable anatomical conditions, particularly when a direct vasal reconstruction is possible and intact sperm or sperm fragments are present in the vasal fluid (5, 27). Conversely, VE is a technically more demanding bypass procedure used when epididymal obstruction is suspected, especially when vasal fluid findings suggest absent sperm or secondary epididymal obstruction (27–29). Thus, lower outcomes after VE may reflect confounding by indication because VE is used for more severe or distal obstruction.

The likelihood of epididymal obstruction and the need for VE increase with longer obstructive interval and have been linked to epididymal blowout or dysfunction after prolonged blockage (28). Therefore, the lower statistical success rates observed after VE should be interpreted as partly reflecting selection into a more severe obstructive phenotype, rather than a simple deficiency of the surgical technique itself.

Intraoperatively, the choice between VV and VE is dictated by vasal fluid analysis; the absence of sperm or presence of thick, paste-like fluid suggests secondary epididymal obstruction caused by chronic backpressure, necessitating VE (20, 26, 30). This rationale supports analyzing mixed bilateral reconstructions as a separate group. Mixed bilateral approaches showed intermediate patency, lower than bilateral VV but higher than bilateral VE, reflecting asymmetric intraoperative findings rather than a therapeutic compromise. This supports unilateral VE when fluid analysis requires it, provided contralateral VV remains feasible. This point is especially important during preoperative counseling for patients with prolonged obstructive intervals, typically >10 to 15 years, who are more likely to require at least unilateral VE (6, 20).

Because the appropriate reconstruction cannot be definitively selected until intraoperative vasal fluid assessment is performed, these findings have an important implication for surgical training. Reproductive urologists performing VR should be proficient in both microsurgical VV and VE. A surgeon limited to VV may be unable to provide the indicated reconstruction when epididymal obstruction is encountered, reducing the patient's chance of fertility restoration (31).

Subgroup analyses based on intraoperative criteria indicate that the higher observed patency after VV is consistent across varying fluid conditions and selection stringencies. Although prior failed reversal may signal complex obstruction, it did not significantly moderate patency or pregnancy in our exploratory metaregression. Thus, intraoperative vasal fluid assessment remains more informative for surgical selection than preoperative history alone.

Pregnancy outcomes were numerically higher after VV but require cautious interpretation because they depend on patency and couple-level factors. Patent-only analyses suggest that greater patency largely accounts for this difference. Among patent patients, natural and overall pregnancy did not differ by technique, although overall live birth remained higher after VV; the overall live birth estimate was based on few studies and should be interpreted cautiously. The pooled odds of natural pregnancy were higher in patients with available reproductive follow-up for VV (OR 2.01), and the overall live birth analysis similarly favored VV (OR 2.79). We hypothesize that the widened advantage of VV in ART-inclusive pregnancy rates (OR 2.92) may stem from selection bias. Couples pursuing ART after VE often do so to bypass persistent patency failures, whereas couples utilizing ART after VV may do so for unrelated female-factor infertility, which generally carries more favorable conception odds. Ultimately, overall reproductive success depends on multiple couple-level factors, notably the female partner's age (20). Exploratory meta-regression further indicated that longer follow-up duration attenuated the VV advantage for natural pregnancy. However, this finding is hypothesis-generating given the limited number of studies and risk of ecological bias. In addition, sperm return occurred earlier after VV, by approximately 2 months. This corresponds to the reported values of time to patency of 1.7 to 4.3 months in VV compared to 2.8 to 6.6 months in VE (9). Overall, patency may explain the initial pregnancy difference, whereas couple-level factors, baseline fertility, and ART use shape outcomes among patent men.

The observed patency and pregnancy differences associated with VV also carry economic implications. Pavlovich and Schlegel reported a cost per live delivery of $25,475 for VR versus $72,521 for IVF, accounting for direct and indirect costs (3). Kolettis and Thomas reported comparable figures of $31,099 versus $51,024 per live delivery (32). In women older than 37 years, Deck and Berger found a cost per live delivery of $28,530 with VR compared with $103,940 with IVF (33). These differences are most pronounced when at least unilateral VV is feasible; when bilateral VE is required, patency rates fall to 54-72%, and the economic case for VR weakens accordingly. Insurance coverage and access to fellowship-trained microsurgeons modify this comparison and should be addressed during preoperative counselling, particularly for patients with prolonged obstructive intervals who are more likely to require VE.

Results from the subgroup analysis by VE technique suggested that intussusception was associated with no heterogeneity compared to moderate heterogeneity for traditional VE techniques. By inserting the smaller epididymis into the vas deferens to create a watertight closure, intussusception minimizes complications like leakage, granuloma formation, and strictures associated with older techniques (26, 34). It may therefore yield more consistent outcomes than end-to-side or end-to-end techniques (7, 35). Neither definition of patency (anatomical or functional) appeared to affect the VV benefit, but the lack of uniform patency criteria still poses problems in the methodology (25). Overall, differences likely reflect case complexity and indication rather than intrinsic technique superiority; technique determines patency, while couple-level factors modify subsequent fertility.

### Strengths and Limitations

This is the first systematic three-way pooled comparison of VV, VE, and mixed procedures, supported by rigorous sensitivity analyses. However, several limitations warrant mention. First, all data came from observational cohorts, and pooled estimates were based on crude, unadjusted odds ratios; important confounders such as female partner age, obstructive interval, vasal fluid quality, and laterality were therefore not captured. Second, substantial statistical heterogeneity (I^2^ > 50%) across several outcomes reduces the reliability of the pooled estimates and underscores the inherent variability in surgical techniques, patient populations, and surgeon expertise. Third, significant loss to follow-up across even the largest cohorts limits the robustness of pooled pregnancy and overall live birth estimates. Fourth, studies did not consistently distinguish ART pregnancies using ejaculated sperm after reversal from those using surgically retrieved sperm; ART-inclusive estimates are therefore exploratory and should not be interpreted as technique-specific efficacy. This same limitation applies to the overall live birth outcome, which combines natural and ART-assisted deliveries across studies with inconsistent reporting of conception mode. Fifth, baseline characteristics regarding previous failed reversals varied widely, and the definitive impact of prior failed reversal on subsequent mixed bilateral procedure outcomes remains difficult to isolate despite exploratory meta-regression. Finally, considerable heterogeneity precluded meaningful interpretation of the pooled late failure estimate, highlighting a critical gap in the tracking of secondary azoospermia. Future research must prioritize prospective registries with standardized follow-up protocols for both patency and couple-level pregnancy outcomes.

## CONCLUSIONS

When intraoperative vasal fluid analysis permits direct anastomosis, microsurgical VV is associated with higher patency, natural pregnancy, and overall live birth rates compared to VE. However, these differences are likely influenced by patient selection and baseline obstruction severity. Conversely, VE remains the essential and appropriate reconstructive procedure when secondary epididymal obstruction is encountered. Patency after mixed bilateral reconstruction is intermediate, accurately reflecting asymmetric intraoperative anatomy rather than a therapeutic compromise. These findings establish intraoperative vasal fluid assessment as the central decision point for matching each vasal unit to the appropriate technique.

## Data Availability

All data generated or analysed during this study are included in this published article

## ABBREVIATIONS

AHRQ: = Agency for Healthcare Research and Quality
ART: = Assisted reproductive technology
CI: = Confidence interval
CIOMS: = Council for International Organizations of Medical Sciences
ICSI: = Intracytoplasmic sperm injection
LIVE: = Longitudinal intussusception
MD: = Mean difference
Mixed: = Bilateral procedure with VV on one side and VE on the other
n: = Number
NOS: = Newcastle-Ottawa Scale
NR: = Not reported
OI: = Obstructive interval (time since vasectomy)
OR: = Odds ratio
PICOS: = Population, Intervention, Comparison, Outcomes, and Study design
PRISMA: = Preferred Reporting Items for Systematic Reviews and Meta-Analyses
SD: = Standard deviation
VE: = Vasoepididymostomy
VR: = Vasectomy reversal
VV: = Vasovasostomy
y: = Years
