# Supplementary material for: Patency, Pregnancy, and Live Birth Outcomes of Microsurgical Vasovasostomy Compared with Vasoepididymostomy for Vasectomy Reversal: A Systematic Review and Meta-Analysis
Source: Int Braz J Urol. 2026 Jul 30;52(6):e20260353. doi: 10.1590/S1677-5538.IBJU.2026.0353 (PMC13682759; doi:10.1590/S1677-5538.IBJU.2026.0353)
Supplement: Supplementary file 1 [file 1677-6119-ibju-52-6-e20260353-Suppl01.pdf]

APPENDIX 1

PubMed

(("Sterilization Reversal"[Mesh] AND "Male"[Mesh]) OR "Vasovasostomy"[Mesh] OR "Vasoepididymostomy"[Mesh] OR vasovasostom*[tiab] OR vaso-vasostom*[tiab] OR "vasectomy reversal"[tiab] OR "vasectomies reversal"[tiab] OR "vas reversal"[tiab] OR vasoepididymostom*[tiab] OR vaso-epididymostom*[tiab] OR epididymovasostom*[tiab] OR epididymo-vasostom*[tiab])

Scopus

(vasovasostom* OR vaso-vasostom* OR "vasectomy reversal" OR "vasectomies reversal" OR "vas reversal" OR "vas deferens reversal" OR "sterilization reversal") AND (vasoepididymostom* OR vaso-epididymostom* OR epididymo-vasostom* OR epididymo-vasostom* OR "vasoepididymal anastomosis")

Epistemonikos

(vasovasostom* OR vaso-vasostom* OR "vasectomy reversal" OR "vasectomies reversal" OR "vas reversal" OR "vas deferens reversal" OR "sterilization reversal" OR vasoepididymostom* OR vaso-epididymostom* OR epididymovasostom* OR epididymo-vasostom* OR "vasoepididymal anastomosis")

Europe PMC

(TITLE_ABS:vasovasostom* OR TITLE_ABS:vaso-vasostom* OR TITLE_ABS:"vasectomy reversal" OR TITLE_ABS:"vasectomies reversal" OR TITLE_ABS:"vas reversal" OR TITLE_ABS:"vas deferens reversal" OR TITLE_ABS:"sterilization reversal" OR TITLE_ABS:vasoepididymostom* OR TITLE_ABS:vaso-epididymostom* OR TITLE_ABS:epididymovasostom* OR TITLE_ABS:epididymo-vasostom* OR TITLE_ABS:"vasoepididymal anastomosis")

APPENDIX 2

CORRECTED RESULTS

Overall Pregnancy Rate

Study	VV Events	VV Total	VE Events	VE Total	Weight	OR [95% CI]
Fuchs_2002	19	40	17	33	18.5%	0.85 [0.34; 2.14]
Magheli_2010	154	234	14	24	20.3%	1.38 [0.58; 3.23]
Matthews_1995	39	98	17	52	25.2%	1.36 [0.67; 2.76]
Schrepferman_2001	26	44	2	9	7.4%	5.06 [0.94; 27.19]
Schwarzer_2004	106	167	19	33	23.4%	1.28 [0.60; 2.73]
Boorjian_2004	132	151	29	30	5.2%	0.24 [0.03; 1.86]
**Total (95% CI)**	**476**	**734**	**98**	**181**	**100.0%**	**1.24 [0.76; 2.03]**

Heterogeneity: $Tau^2 = 0.1228$; $Chi^2 = 5.92$, df = 5 (P = 0.3146); $I^2 = 15.5\%$

Test for overall effect: Z = 0.85 (P = 0.3927)

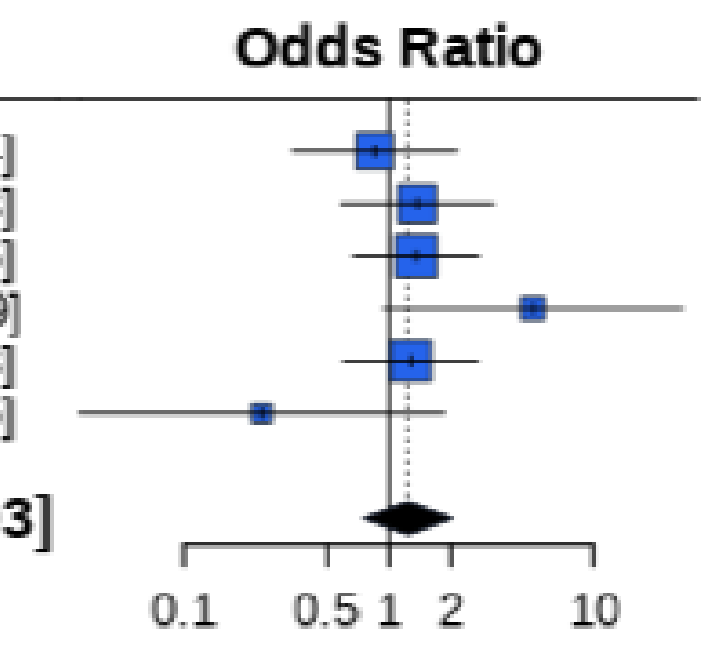

EXCLUDED NON PATENT

Natural pregnancy

Study	VV Events	VV Total	VE Events	VE Total	Weight	OR [95% CI]
Fuchs_2002	19	40	17	33	18.5%	0.85 [0.34; 2.14]
Magheli_2010	154	234	14	24	20.3%	1.38 [0.58; 3.23]
Matthews_1995	39	98	17	52	25.2%	1.36 [0.67; 2.76]
Schrepferman_2001	26	44	2	9	7.4%	5.06 [0.94; 27.19]
Schwarzer_2004	106	167	19	33	23.4%	1.28 [0.60; 2.73]
Boorjian_2004	132	151	29	30	5.2%	0.24 [0.03; 1.86]
**Total (95% CI)**	**476**	**734**	**98**	**181**	**100.0%**	**1.24 [0.76; 2.03]**

Heterogeneity: $Tau^2 = 0.1228$; $Chi^2 = 5.92$, df = 5 (P = 0.3146); $I^2 = 15.5\%$
Test for overall effect: Z = 0.85 (P = 0.3927)

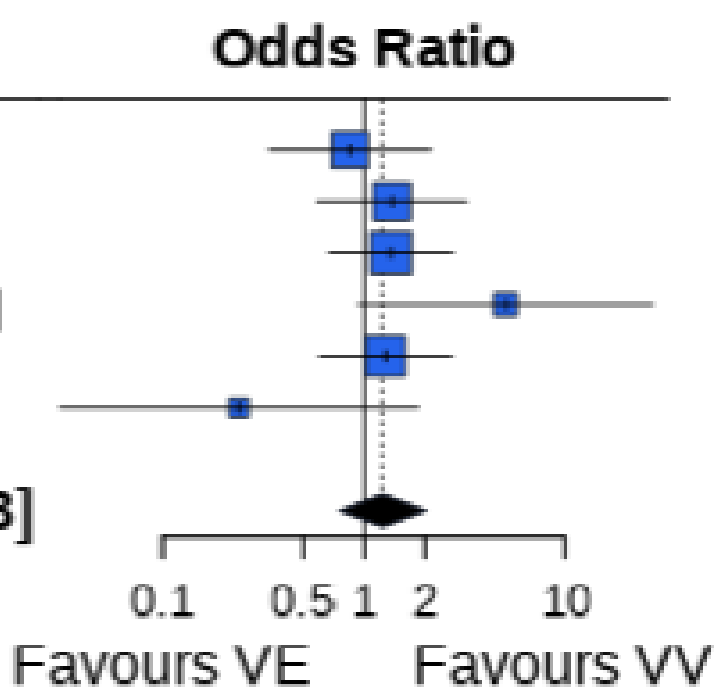

Overall pregnancy

Study	VV Events	VV Total	VE Events	VE Total	Weight	OR [95% CI]
Matthews_1995	52	98	21	52	33.8%	1.67 [0.84; 3.30]
Silber_2004	639	685	296	339	43.5%	2.02 [1.30; 3.13]
Boorjian_2004	132	151	29	30	8.3%	0.24 [0.03; 1.86]
Matthews_1997	12	26	4	11	14.4%	1.50 [0.35; 6.40]
**Total (95% CI)**	**835**	**960**	**350**	**432**	**100.0%**	**1.52 [0.80; 2.89]**

Heterogeneity: $Tau^2 = 0.1958$; $Chi^2 = 4.08$, df = 3 (P = 0.2533); $I^2 = 26.4\%$
Test for overall effect: Z = 1.28 (P = 0.2007)

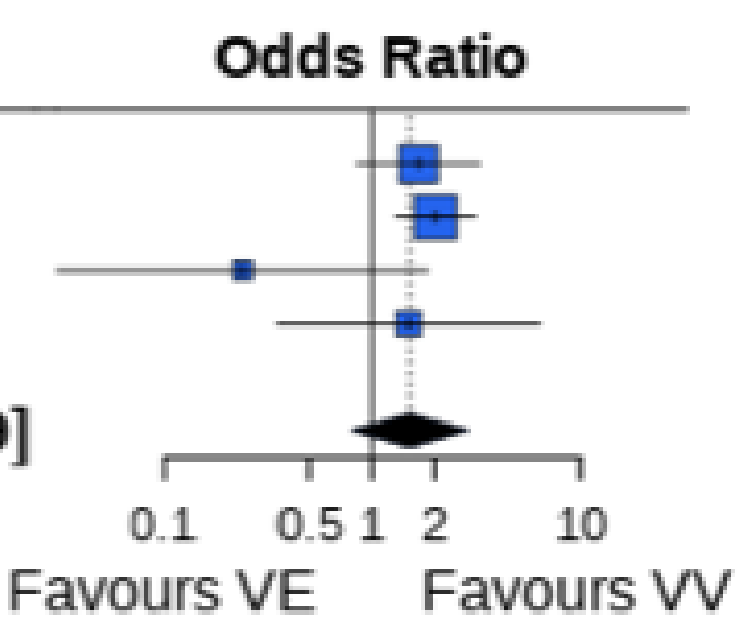

Live birth rate

Study	VV Events	VV Total	VE Events	VE Total	Weight	OR [95% CI]
Matthews_1995	52	98	21	52	23.1%	1.67 [0.84; 3.30]
Silber_2004	419	490	193	251	71.8%	1.77 [1.20; 2.61]
Matthews_1997	12	26	4	11	5.1%	1.50 [0.35; 6.40]
**Total (95% CI)**	**483**	**614**	**218**	**314**	**100.0%**	**1.73 [1.25; 2.41]**

Heterogeneity: $Tau^2 = 0$; $Chi^2 = 0.06$, df = 2 (P = 0.9687); $I^2 = 0.0\%$
Test for overall effect: Z = 3.29 (P = 0.0010)

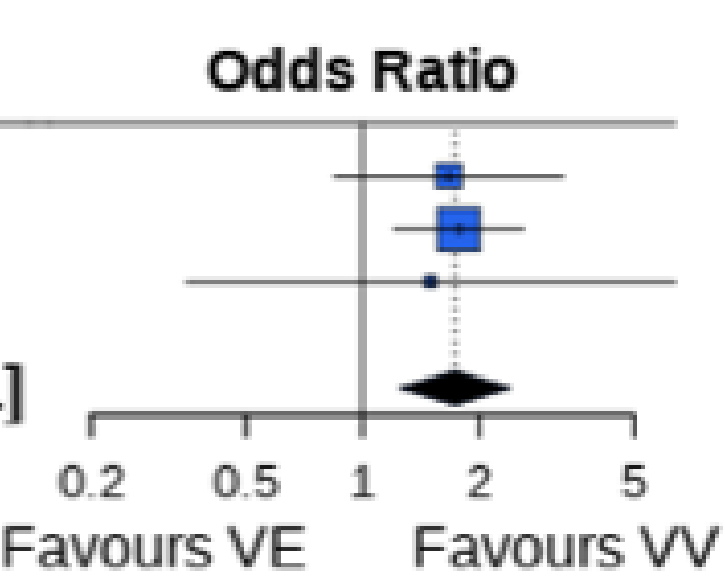

ALL META REGRESSIONS ARE EXPLORATORY, LACK OF FOLLOW UP REPORTING

Meta-regress FEMALE AGE

Natural pregnancy

Overall pregnancy and live birth = cant regress, due to moderator availability mismatch and/or low k

Regression Statistics

```
Mixed-Effects Model (k = 4; tau^2 estimator: PM)

tau^2 (estimated amount of residual heterogeneity):     0 (SE = 0.0941)
tau (square root of estimated tau^2 value):             0
I^2 (residual heterogeneity / unaccounted variability): 0.00%
H^2 (unaccounted variability / sampling variability):   1.00
R^2 (amount of heterogeneity accounted for):            100.00%

Test for Residual Heterogeneity:
QE(df = 2) = 1.8540, p-val = 0.3957

Test of Moderators (coefficient 2):
QM(df = 1) = 4.9273, p-val = 0.0264

Model Results:

                                      estimate      se     zval    pval    ci.lb    ci.ub
intrcpt                                 9.6185  4.0020   2.4034  0.0162   1.7748  17.4622  *
Mean_Age_of_Female_Partner_moderator   -0.2832  0.1276  -2.2198  0.0264  -0.5332  -0.0331  *

---
Signif. codes:  0 '***' 0.001 '**' 0.01 '*' 0.05 '.' 0.1 ' ' 1
```

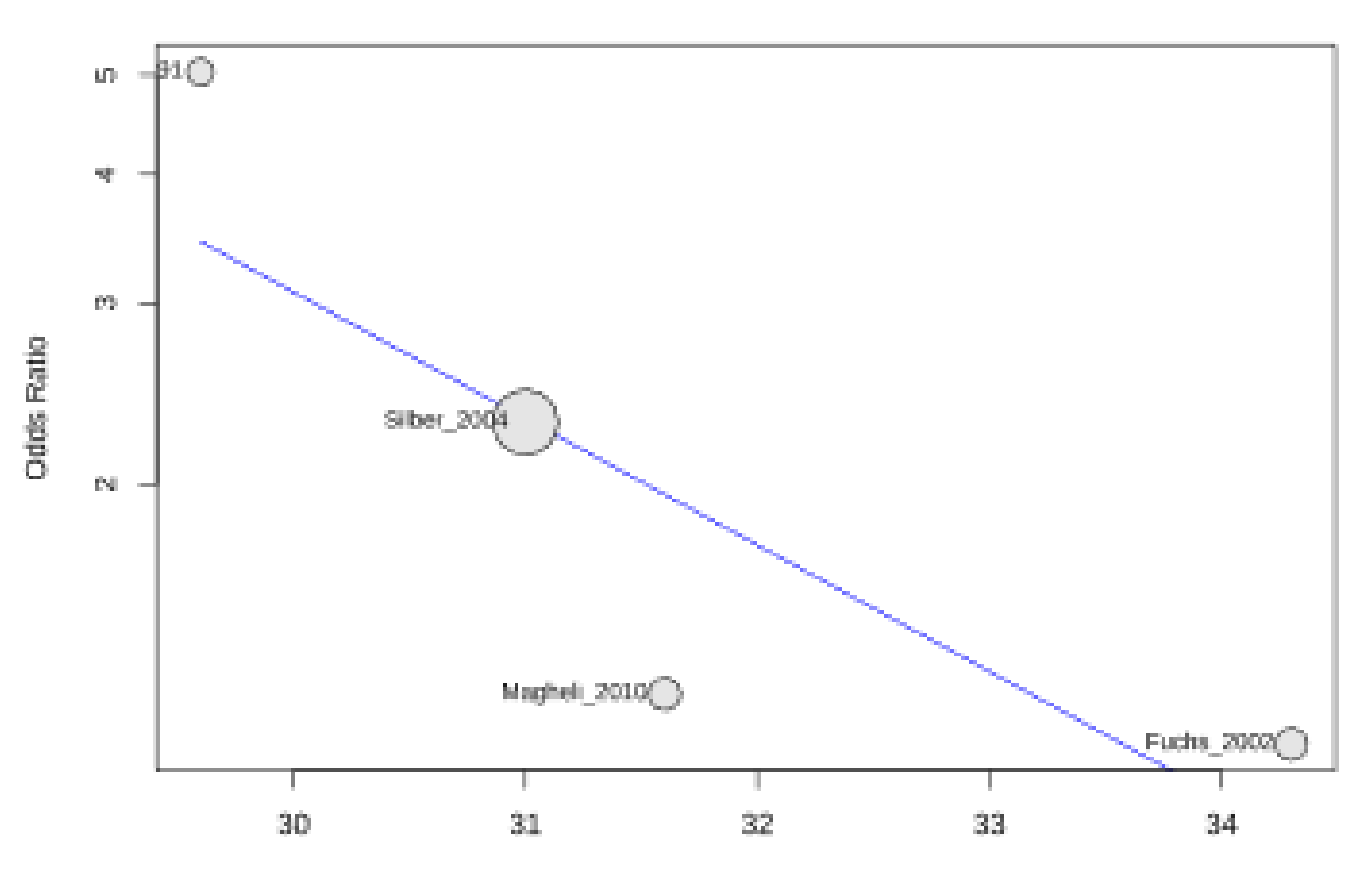

Meta-regress FOLLOW UP

Natural pregnancy

Regression Statistics

```
Mixed-Effects Model (k = 5; tau^2 estimator: PM)

tau^2 (estimated amount of residual heterogeneity):     0 (SE = 0.0807)
tau (square root of estimated tau^2 value):             0
I^2 (residual heterogeneity / unaccounted variability): 0.00%
H^2 (unaccounted variability / sampling variability):   1.00
R^2 (amount of heterogeneity accounted for):            100.00%

Test for Residual Heterogeneity:
QE(df = 3) = 2.0529, p-val = 0.5615

Test of Moderators (coefficient 2):
QM(df = 1) = 5.9327, p-val = 0.0149

Model Results:

         estimate      se     zval    pval    ci.lb    ci.ub
intrcpt    2.2745  0.6159   3.6928  0.0002   1.0673   3.4817  ***
FU        -0.0856  0.0351  -2.4357  0.0149  -0.1545  -0.0167    *

---
Signif. codes:  0 '***' 0.001 '**' 0.01 '*' 0.05 '.' 0.1 ' ' 1
```

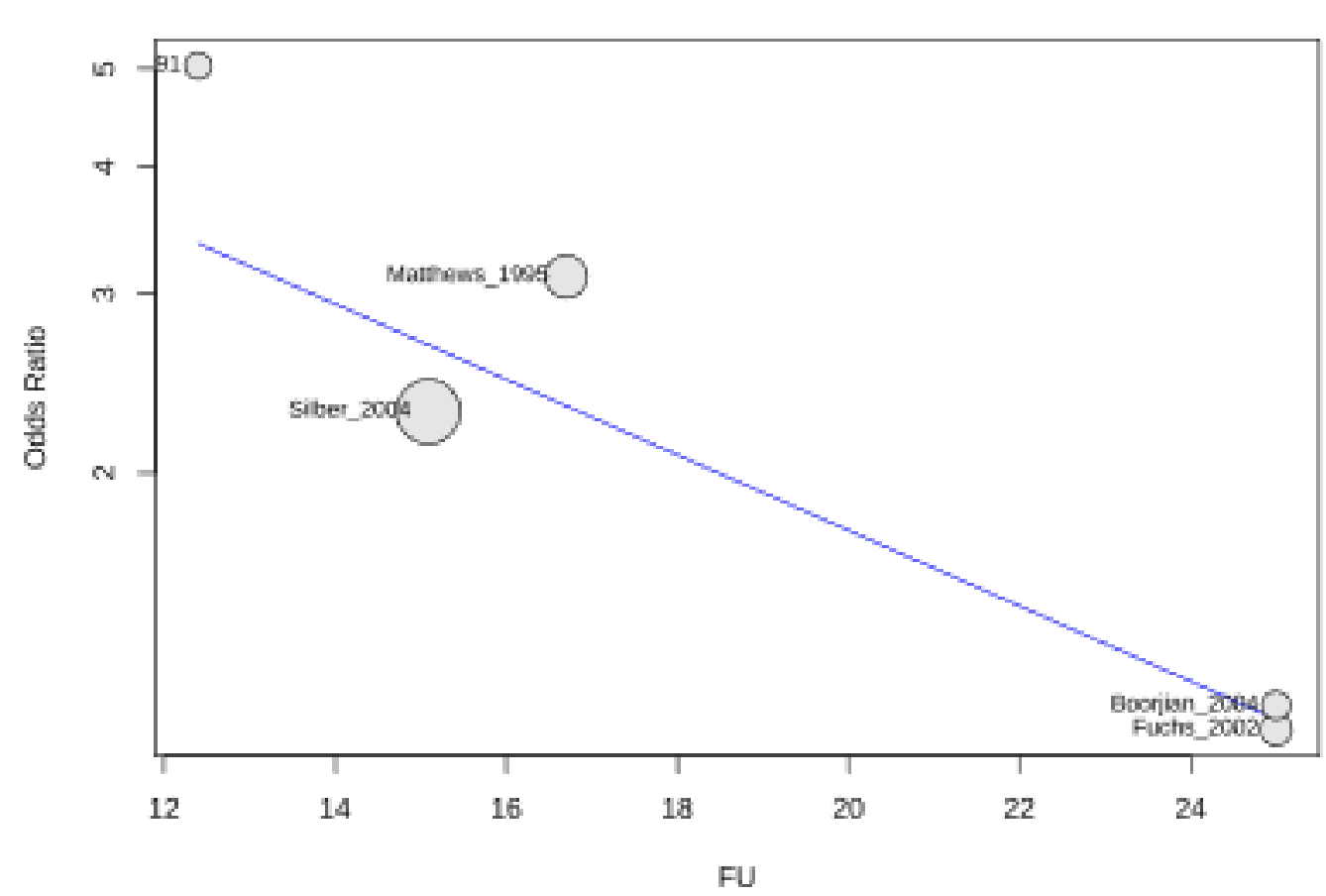

Overall pregnancy

Regression Statistics

```
Mixed-Effects Model (k = 5; tau^2 estimator: PM)

tau^2 (estimated amount of residual heterogeneity):     0.1190 (SE = 0.2172)
tau (square root of estimated tau^2 value):             0.3450
I^2 (residual heterogeneity / unaccounted variability): 49.26%
H^2 (unaccounted variability / sampling variability):   1.97
R^2 (amount of heterogeneity accounted for):            30.60%

Test for Residual Heterogeneity:
QE(df = 3) = 6.4095, p-val = 0.0933

Test of Moderators (coefficient 2):
QM(df = 1) = 1.6919, p-val = 0.1934

Model Results:

         estimate      se     zval    pval    ci.lb   ci.ub
intrcpt    2.3085  0.9834   2.3475  0.0189   0.3811  4.2360  *
FU        -0.0719  0.0553  -1.3007  0.1934  -0.1803  0.0365

---
Signif. codes:  0 '***' 0.001 '**' 0.01 '*' 0.05 '.' 0.1 ' ' 1
```

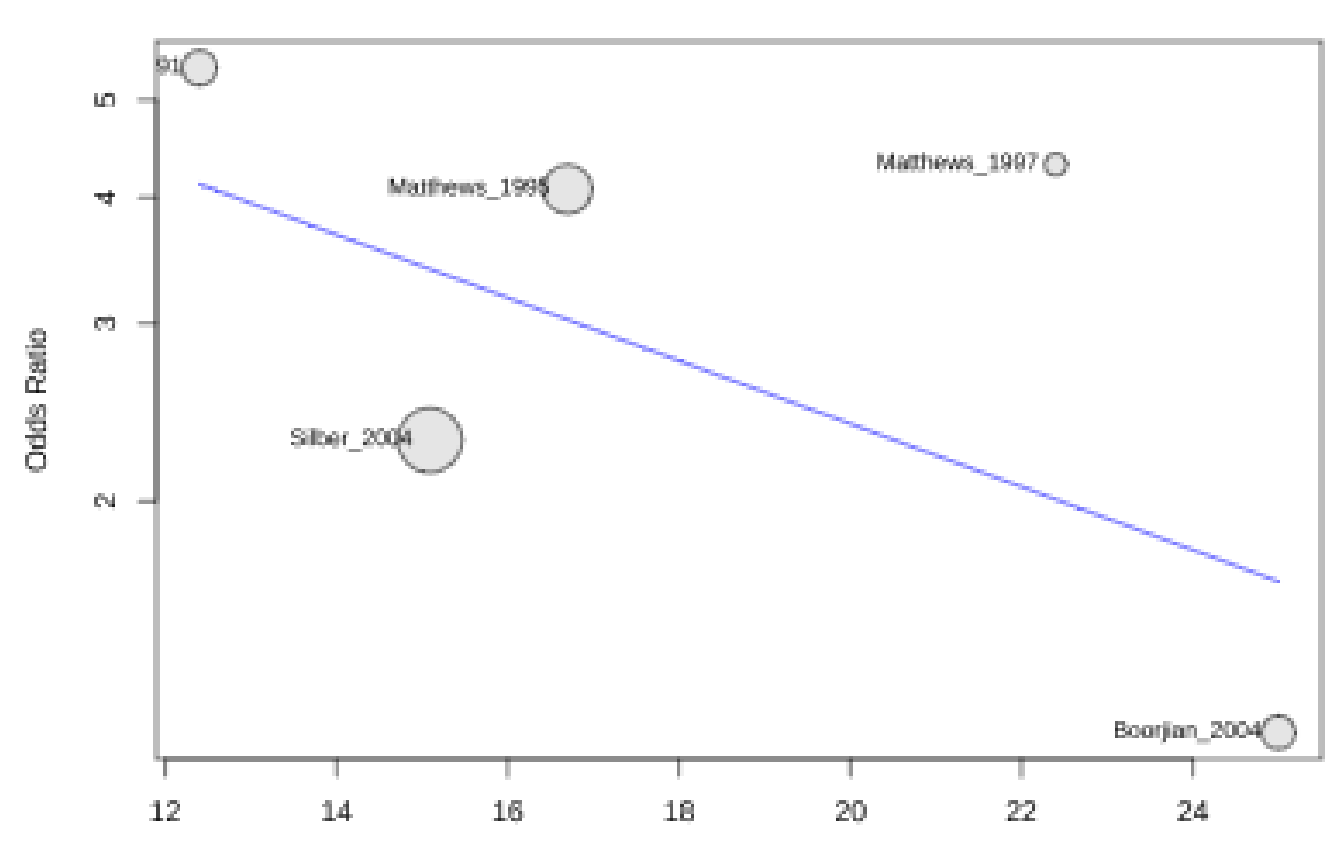

Live birth

Regression Statistics

```
Mixed-Effects Model (k = 3; tau^2 estimator: PM)

tau^2 (estimated amount of residual heterogeneity):     0.1008 (SE = 0.2929)
tau (square root of estimated tau^2 value):             0.3175
I^2 (residual heterogeneity / unaccounted variability): 54.44%
H^2 (unaccounted variability / sampling variability):   2.19
R^2 (amount of heterogeneity accounted for):            0.00%

Test for Residual Heterogeneity:
QE(df = 1) = 2.1949, p-val = 0.1385

Test of Moderators (coefficient 2):
QM(df = 1) = 0.9888, p-val = 0.3200

Model Results:

         estimate      se     zval    pval    ci.lb   ci.ub
intrcpt   -0.8173  1.8743  -0.4360  0.6628  -4.4909  2.8563
FU         0.1113  0.1120   0.9944  0.3200  -0.1081  0.3307

---
Signif. codes:  0 '***' 0.001 '**' 0.01 '*' 0.05 '.' 0.1 ' ' 1
```

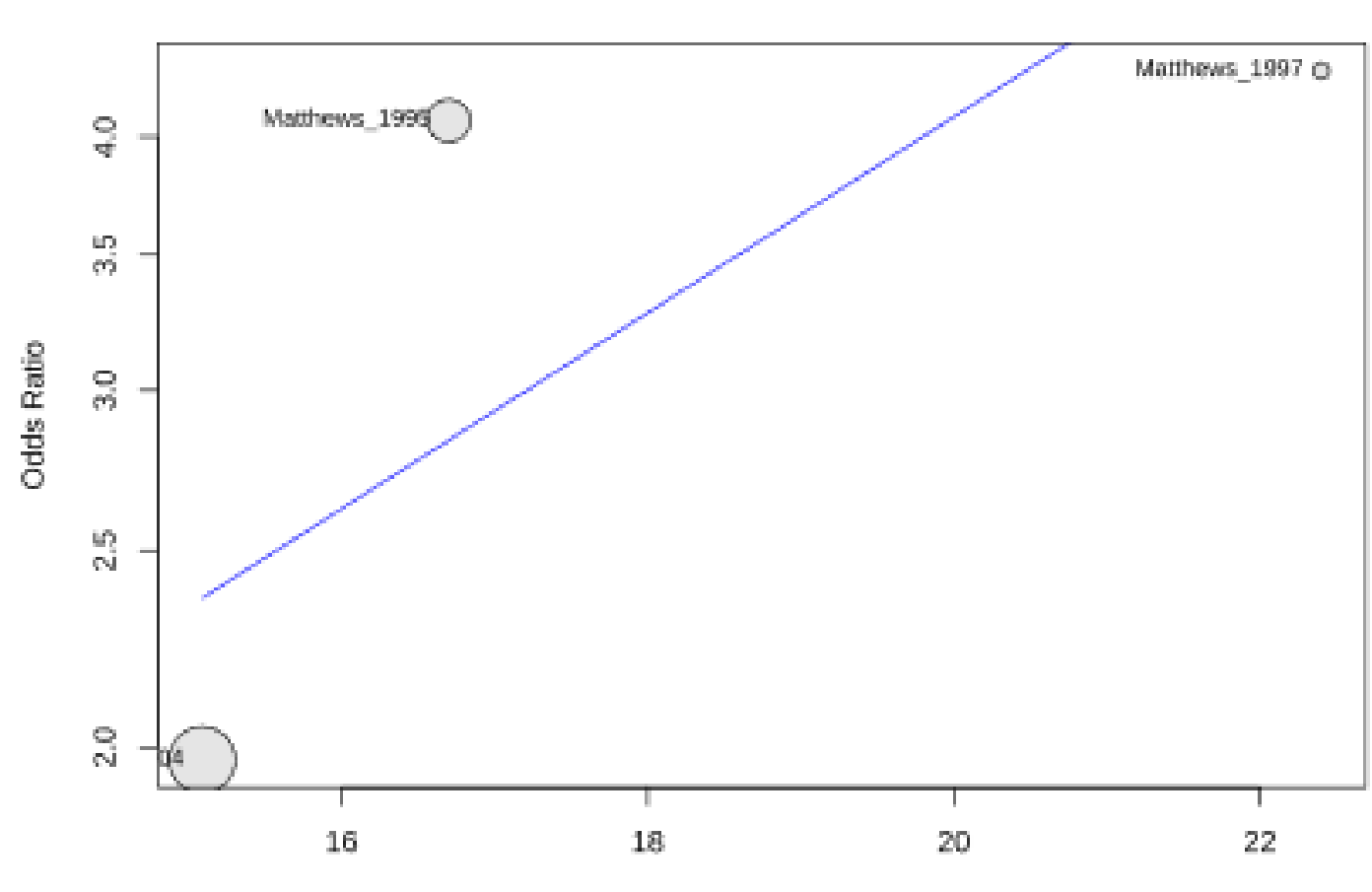

META-REGRESS FU PATENCY

VV vs VE

Regression Statistics

```
Mixed-Effects Model (k = 4; tau^2 estimator: PM)

tau^2 (estimated amount of residual heterogeneity):     0.6882 (SE = 1.2650)
tau (square root of estimated tau^2 value):             0.8296
I^2 (residual heterogeneity / unaccounted variability): 50.80%
H^2 (unaccounted variability / sampling variability):   2.03
R^2 (amount of heterogeneity accounted for):            0.00%

Test for Residual Heterogeneity:
QE(df = 2) = 4.1872, p-val = 0.1232

Test of Moderators (coefficient 2):
QM(df = 1) = 0.7453, p-val = 0.3880

Model Results:

            estimate      se     zval    pval    ci.lb   ci.ub
intrcpt       4.3816  2.2053   1.9868  0.0469   0.0592  8.7040  *
FU_patency   -0.0938  0.1086  -0.8633  0.3880  -0.3066  0.1191

---
Signif. codes:  0 '***' 0.001 '**' 0.01 '*' 0.05 '.' 0.1 ' ' 1
```

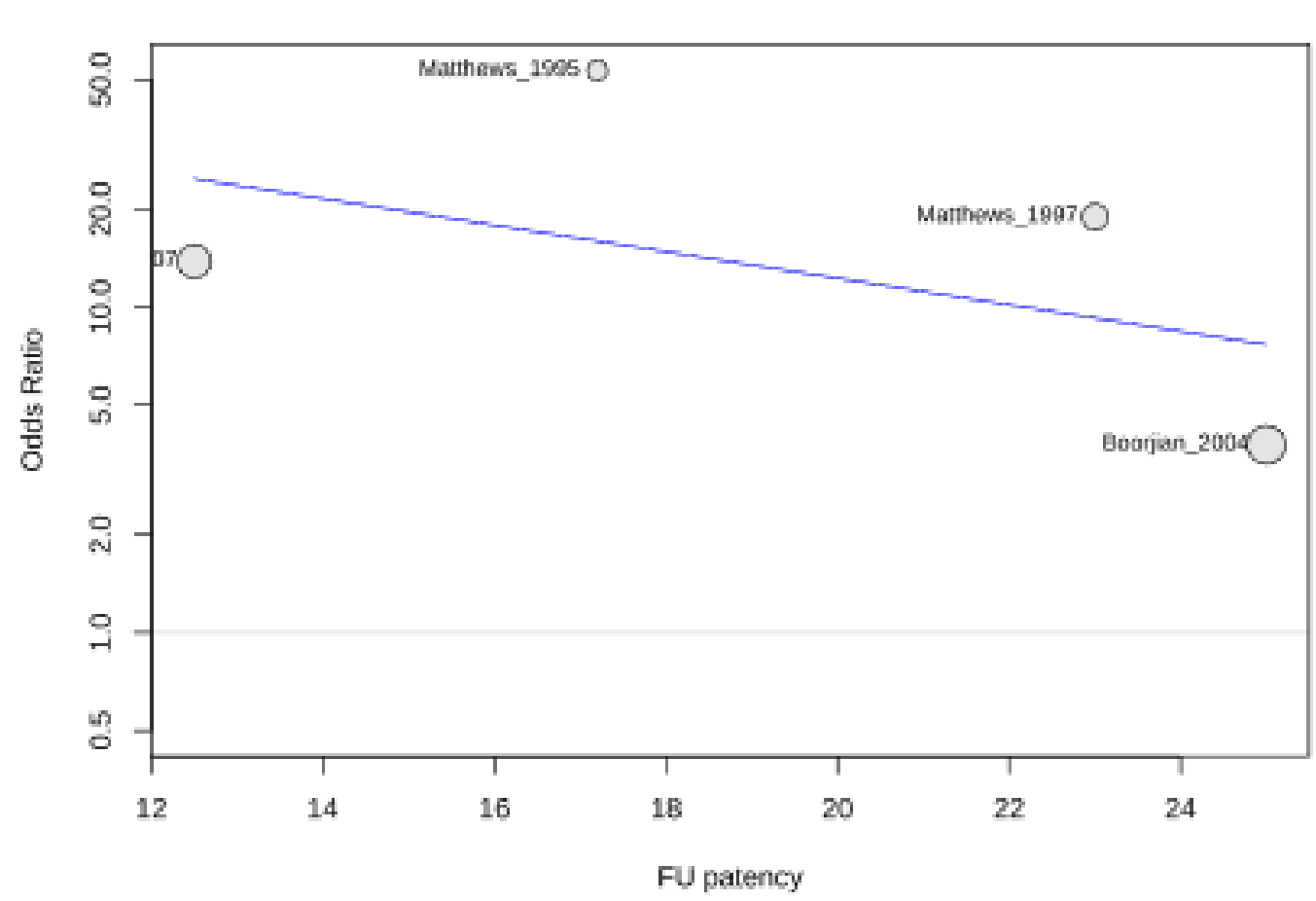

VV vs Mixed, VE vs Mixed = cant regress, due to moderator availability mismatch and/or low k

META-REGRESS PREVIOUS FAILED REVERSAL %

Patency VV vs VE

Regression Statistics

```
Mixed-Effects Model (k = 7; tau^2 estimator: PM)

tau^2 (estimated amount of residual heterogeneity):     0.1019 (SE = 0.1659)
tau (square root of estimated tau^2 value):             0.3192
I^2 (residual heterogeneity / unaccounted variability): 46.13%
H^2 (unaccounted variability / sampling variability):   1.86
R^2 (amount of heterogeneity accounted for):            18.82%

Test for Residual Heterogeneity:
QE(df = 5) = 9.6832, p-val = 0.0847

Test of Moderators (coefficient 2):
QM(df = 1) = 1.4708, p-val = 0.2252

Model Results:

                            estimate      se     zval     pval    ci.lb    ci.ub
intrcpt                       1.5323  0.2623   5.8411   <.0001   1.0182   2.0465   ***
Previous_Failed_Reversal__    0.0120  0.0099   1.2128   0.2252  -0.0074   0.0314

---
Signif. codes:  0 '***' 0.001 '**' 0.01 '*' 0.05 '.' 0.1 ' ' 1
```

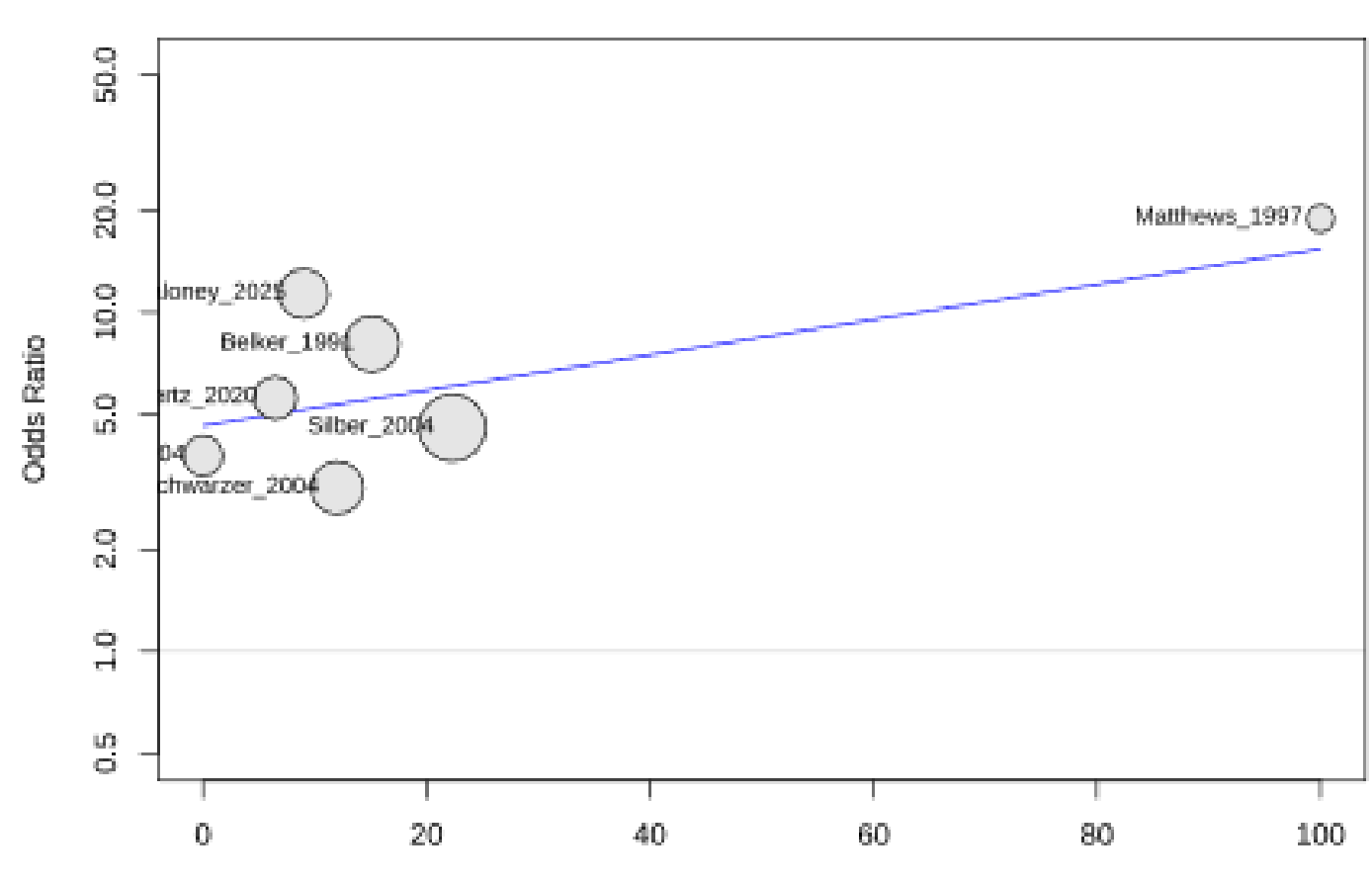

Patency VV vs Mixed

Regression Statistics

```
Mixed-Effects Model (k = 7; tau^2 estimator: PM)

tau^2 (estimated amount of residual heterogeneity):     0.3913 (SE = 0.4224)
tau (square root of estimated tau^2 value):             0.6255
I^2 (residual heterogeneity / unaccounted variability): 72.75%
H^2 (unaccounted variability / sampling variability):   3.67
R^2 (amount of heterogeneity accounted for):            0.00%

Test for Residual Heterogeneity:
QE(df = 5) = 15.2530, p-val = 0.0093

Test of Moderators (coefficient 2):
QM(df = 1) = 0.5612, p-val = 0.4538

Model Results:

                            estimate      se    zval    pval    ci.lb   ci.ub
intrcpt                       0.4671  0.3999  1.1681  0.2428  -0.3167  1.2510
Previous_Failed_Reversal__    0.0102  0.0136  0.7492  0.4538  -0.0165  0.0368

---
Signif. codes:  0 '***' 0.001 '**' 0.01 '*' 0.05 '.' 0.1 ' ' 1
```

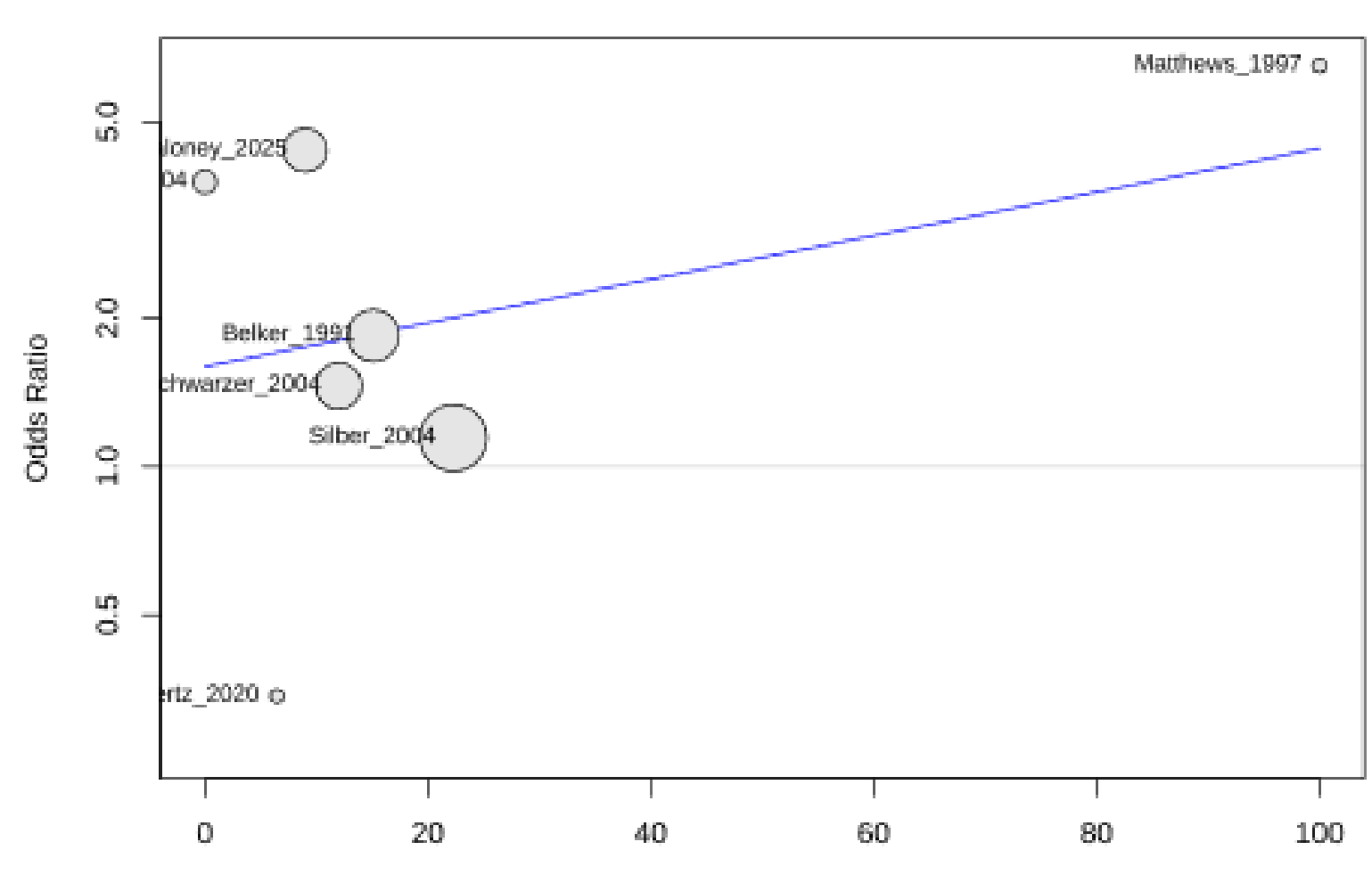

Patency VE vs Mixed

Regression Statistics

```
Mixed-Effects Model (k = 7; tau^2 estimator: PM)

tau^2 (estimated amount of residual heterogeneity):     0.1262 (SE = 0.2336)
tau (square root of estimated tau^2 value):             0.3552
I^2 (residual heterogeneity / unaccounted variability): 41.19%
H^2 (unaccounted variability / sampling variability):   1.70
R^2 (amount of heterogeneity accounted for):            0.00%

Test for Residual Heterogeneity:
QE(df = 5) = 7.0340, p-val = 0.2181

Test of Moderators (coefficient 2):
QM(df = 1) = 0.0541, p-val = 0.8161

Model Results:

                            estimate      se     zval    pval    ci.lb    ci.ub
intrcpt                      -1.1112  0.3119  -3.5627  0.0004  -1.7225  -0.4999  ***
Previous_Failed_Reversal__   -0.0024  0.0102  -0.2326  0.8161  -0.0225   0.0177

---
Signif. codes:  0 '***' 0.001 '**' 0.01 '*' 0.05 '.' 0.1 ' ' 1
```

Natural pregnancy

Regression Statistics

```
Mixed-Effects Model (k = 4; tau^2 estimator: PM)

tau^2 (estimated amount of residual heterogeneity):     0.1412 (SE = 0.2672)
tau (square root of estimated tau^2 value):             0.3757
I^2 (residual heterogeneity / unaccounted variability): 49.87%
H^2 (unaccounted variability / sampling variability):   1.99
R^2 (amount of heterogeneity accounted for):            0.00%

Test for Residual Heterogeneity:
QE(df = 2) = 3.4612, p-val = 0.1772

Test of Moderators (coefficient 2):
QM(df = 1) = 0.9274, p-val = 0.3355

Model Results:

                            estimate      se    zval    pval    ci.lb   ci.ub
intrcpt                       0.3452  0.5290  0.6524  0.5141  -0.6917  1.3821
Previous_Failed_Reversal__    0.0313  0.0325  0.9630  0.3355  -0.0324  0.0949

---
Signif. codes:  0 ‘***’ 0.001 ‘**’ 0.01 ‘*’ 0.05 ‘.’ 0.1 ‘ ’ 1
```

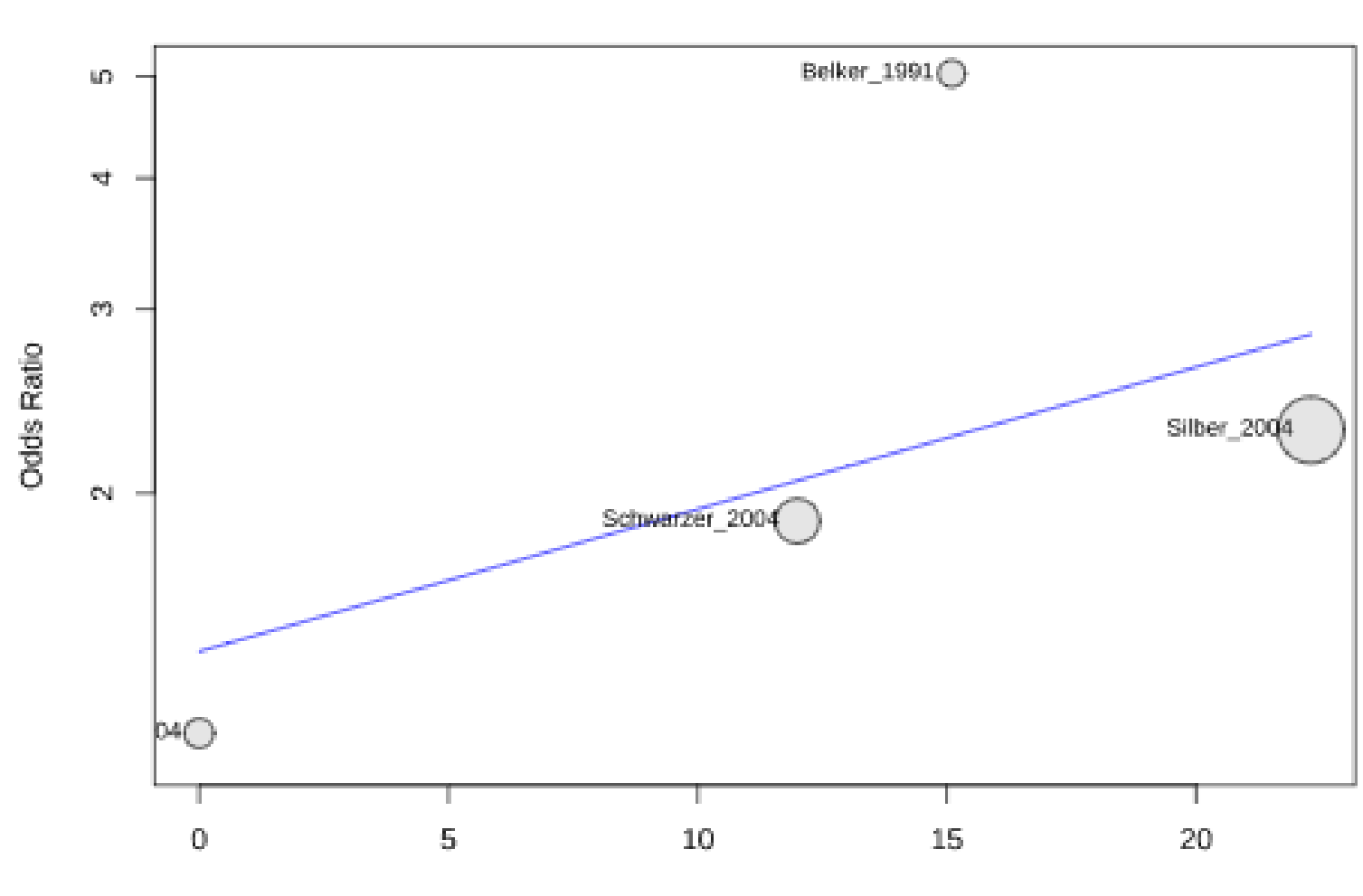

Overall pregnancy

Regression Statistics

```
Mixed-Effects Model (k = 4; tau^2 estimator: PM)

tau^2 (estimated amount of residual heterogeneity):     0.2957 (SE = 0.4824)
tau (square root of estimated tau^2 value):             0.5438
I^2 (residual heterogeneity / unaccounted variability): 70.07%
H^2 (unaccounted variability / sampling variability):   3.34
R^2 (amount of heterogeneity accounted for):            0.00%

Test for Residual Heterogeneity:
QE(df = 2) = 4.8841, p-val = 0.0870

Test of Moderators (coefficient 2):
QM(df = 1) = 0.6388, p-val = 0.4242

Model Results:

                            estimate      se    zval    pval    ci.lb   ci.ub
intrcpt                       0.7463  0.4567  1.6341  0.1022  -0.1488  1.6414
Previous_Failed_Reversal__    0.0084  0.0105  0.7992  0.4242  -0.0122  0.0291

---
Signif. codes:  0 '***' 0.001 '**' 0.01 '*' 0.05 '.' 0.1 ' ' 1
```

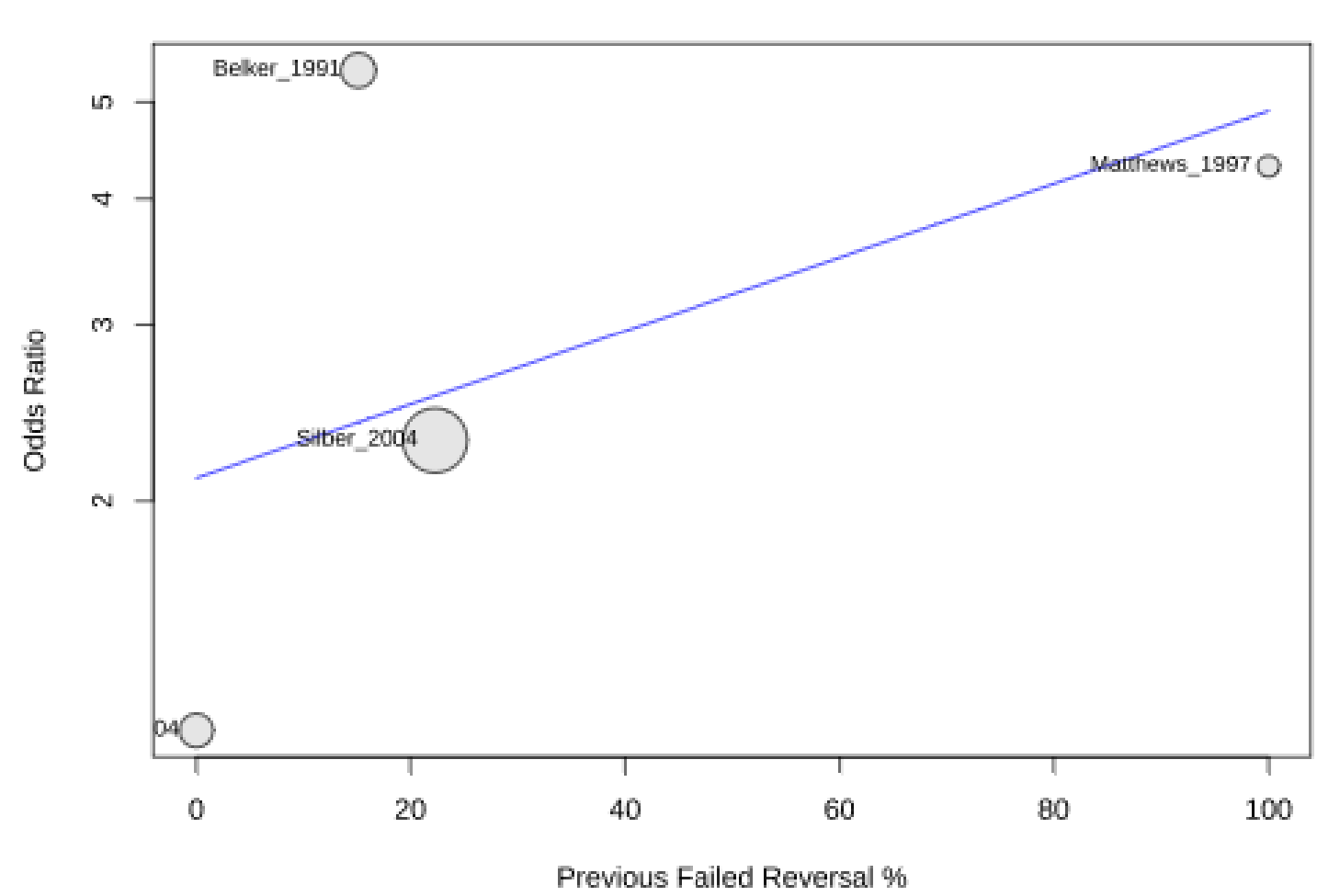

Live birth cant regress low k

NEW SUBGROUP INTRAOPERATIVE CRITERIA

Patency outcomes VV vs VE

Study or Subgroup	VV Events	VV Total	VE Events	VE Total	Weight	OR [95% CI]
**Sperm Quality & Fluid-Dependent Selection (Differentiates between intact sperm vs. sperm fragments/heads)**						
Alhamam_2024	100	101	10	12	3.2%	20.00 [1.66; 240.48]
Maloney_2025	229	248	17	33	10.4%	11.34 [4.96; 25.95]
Silber_2004	1281	1357	797	1013	13.9%	4.57 [3.47; 6.02]
Yang_2007	86	90	14	23	7.3%	13.82 [3.74; 51.03]
**Total (95% CI)**	**1696**	**1796**	**838**	**1081**	**34.7%**	**7.55 [4.13; 13.78]**
Heterogeneity: $Tau^2 = 0.1634$; $Chi^2 = 7.52$, df = 3 (P = 0.0569); $I^2 = 60.1\%$						
Test for overall effect: Z = 6.58 (P < 0.0001)						
**Liberal VV Selection (VV performed despite poor or absent sperm)**						
Belker_1991	920	1061	17	38	11.5%	8.06 [4.15; 15.65]
**Strict Azoospermia / Absence of Intact Sperm Selection**						
Fuchs_2002	40	44	33	42	7.5%	2.73 [0.77; 9.66]
Magheli_2010	233	242	24	24	2.5%	0.50 [0.03; 8.88]
Matthews_1995	99	100	65	100	4.3%	53.31 [7.13; 398.75]
Schrepferman_2001	44	46	9	18	5.4%	22.00 [4.05; 119.43]
Schwarzer_2004	167	192	33	48	11.0%	3.04 [1.45; 6.37]
Boorjian_2004	151	159	30	36	8.3%	3.77 [1.22; 11.67]
Matthews_1997	26	28	11	27	5.7%	18.91 [3.70; 96.54]
**Total (95% CI)**	**760**	**811**	**205**	**295**	**44.8%**	**6.36 [2.24; 18.08]**
Heterogeneity: $Tau^2 = 1.3396$; $Chi^2 = 16.11$, df = 6 (P = 0.0132); $I^2 = 62.8\%$						
Test for overall effect: Z = 3.47 (P = 0.0005)						
**Unstandardized / Insufficient Data**						
Hertz_2020	338	385	9	16	8.9%	5.59 [1.99; 15.73]
**Total (95% CI)**	**3714**	**4053**	**1069**	**1430**	**100.0%**	**6.98 [4.23; 11.50]**
Heterogeneity: $Tau^2 = 0.4481$; $Chi^2 = 25.30$, df = 12 (P = 0.0135); $I^2 = 52.6\%$						
Test for overall effect: Z = 7.62 (P < 0.0001)						
Test for subgroup differences: $Chi^2 = 0.42$, df = 3 (P = 0.9367)						

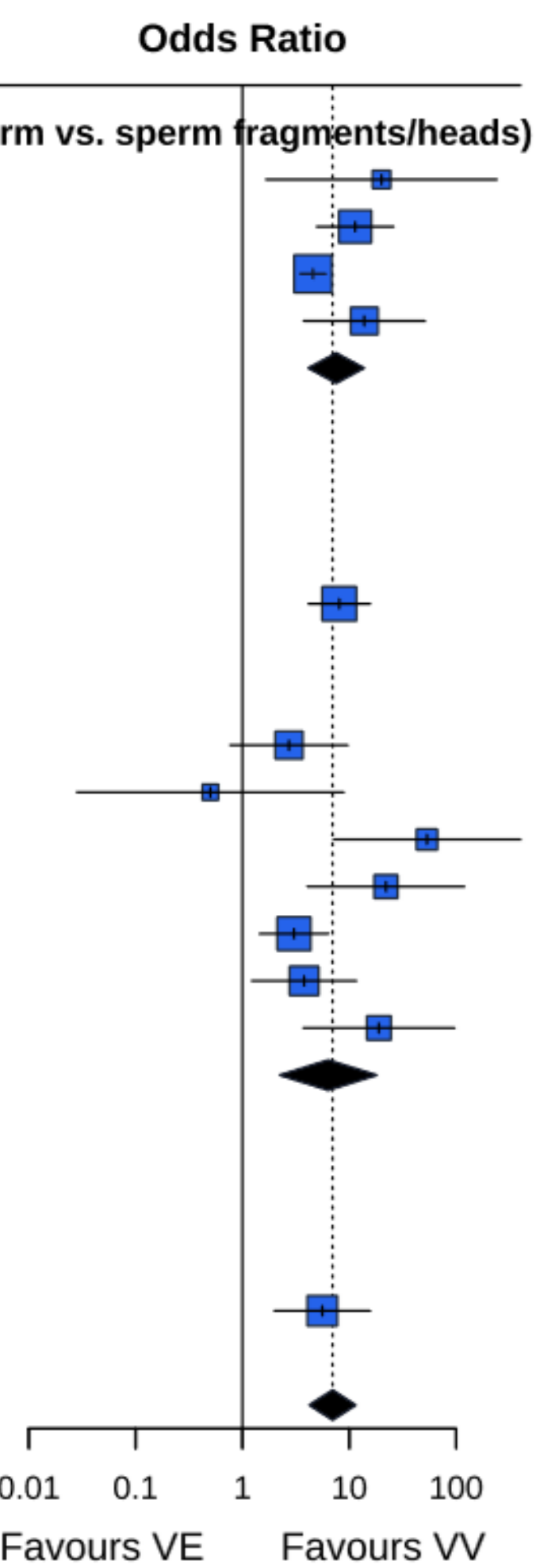

Supplementary Table 1 - Attrition cascade and missing data breakdown for reproductive outcomes by surgical group.

Outcome	Surgical Group	Originally Operated (n)	Achieved Patency (n)	Available Follow-up for Outcome (n)	Number of Events (n)	Missing Outcome Data (%)*
Natural Pregnancy	VV	3,735	2,891	2,372	1,636	36.5%
	VE	1,461	999	704	461	51.8%
Overall Live Birth	VV	1,709	1,406	703	551	58.9%
	VE	1,266	873	443	258	65.0%

* Missing Outcome Data (%) is calculated as: ((Originally Operated - Available Follow-up) / Originally Operated) * 100.

Supplementary Table 2 - Natural pregnancy data verification breakdown.

Study	VV Originally Operated	VE Originally Operated	VV Achieved Patency	VE Achieved Patency	VV Available Follow-up	VE Available Follow-up	VV Number of Events	VE Number of Events
Belker 1991	1,287	40	920	17	834	27	445	5
Fuchs 2002	44	42	40	33	44	42	19	17
Magheli 2010	242	24	233	24	242	24	154	14
Matthews 1995	100	100	99	65	100	100	39	17
Schwarzer 2004	322	80	167	33	192	48	106	19
Silber 2004	1,581	1,139	1,281	797	801	427	741	360
Boorjian 2004	159	36	151	30	159	36	132	29
**TOTAL**	**3,735**	**1,461**	**2,891**	**999**	**2,372**	**704**	**1636**	**461**

Supplementary Table 3 - Overall live birth data verification breakdown.

Study	VV Originally Operated	VE Originally Operated	VV Achieved Patency	VE Achieved Patency	VV Available Follow-up	VE Available Follow-up	VV Number of Events	VE Number of Events
Matthews 1995	100	100	99	65	100	100	52	21
Silber 2004	1,581	1,139	1,281	797	575	316	487	233
Matthews 1997	28	27	26	11	28	27	12	4
**TOTAL**	**1,709**	**1,266**	**1,406**	**873**	**703**	**443**	**551**	**258**
